# Cloning and characterization of bifunctional enzyme farnesyl diphosphate/geranylgeranyl diphosphate synthase from *Plasmodium falciparum*

**DOI:** 10.1186/1475-2875-12-184

**Published:** 2013-06-04

**Authors:** Fabiana M Jordão, Heloisa B Gabriel, João MP Alves, Claudia B Angeli, Thaís D Bifano, Ardala Breda, Mauro F de Azevedo, Luiz A Basso, Gerhard Wunderlich, Emilia A Kimura, Alejandro M Katzin

**Affiliations:** 1 Department of Parasitology, Institute of Biomedical Sciences, University of São Paulo, Av. Lineu Prestes 1374, CEP 05508-000, São Paulo, SP, Brazil; 2 Research Center for Molecular Biology and Functional, National Institute of Science and Technology on Tuberculosis, Pontifical Catholic University of Rio Grande do Sul, Rio Grande do Sul, Brazil; 3 The Macfarlane Burnet Institute for Medical Research and Public Health, Melbourne, Victoria, Australia

**Keywords:** *Plasmodium falciparum*, Malaria, Isoprenoids, Farnesyl diphosphate, Farnesyl diphosphate synthase, Geranylgeranyl diphosphate, Geranylgeranyl diphosphate synthase

## Abstract

**Background:**

Isoprenoids are the most diverse and abundant group of natural products. In *Plasmodium falciparum*, isoprenoid synthesis proceeds through the methyl erythritol diphosphate pathway and the products are further metabolized by farnesyl diphosphate synthase (FPPS), turning this enzyme into a key branch point of the isoprenoid synthesis. Changes in FPPS activity could alter the flux of isoprenoid compounds downstream of FPPS and, hence, play a central role in the regulation of a number of essential functions in *Plasmodium* parasites.

**Methods:**

The isolation and cloning of gene PF3D7_18400 was done by amplification from cDNA from mixed stage parasites of *P*. *falciparum*. After sequencing, the fragment was subcloned in pGEX2T for recombinant protein expression. To verify if the PF3D7_1128400 gene encodes a functional rPfFPPS protein, its catalytic activity was assessed using the substrate [4-^14^C] isopentenyl diphosphate and three different allylic substrates: dimethylallyl diphosphate, geranyl diphosphate or farnesyl diphosphate. The reaction products were identified by thin layer chromatography and reverse phase high-performance liquid chromatography. To confirm the product spectrum formed of rPfFPPS, isoprenic compounds were also identified by mass spectrometry. Apparent kinetic constants *K*_*M*_ and *V*_*max*_ for each substrate were determined by Michaelis–Menten; also, inhibition assays were performed using risedronate.

**Results:**

The expressed protein of *P*. *falciparum* FPPS (rPfFPPS) catalyzes the synthesis of farnesyl diphosphate, as well as geranylgeranyl diphosphate, being therefore a bifunctional FPPS/geranylgeranyl diphosphate synthase (GGPPS) enzyme. The apparent *K*_*M*_ values for the substrates dimethylallyl diphosphate, geranyl diphosphate and farnesyl diphosphate were, respectively, 68 ± 5 μM, 7.8 ± 1.3 μM and 2.06 ± 0.4 μM. The protein is expressed constitutively in all intra-erythrocytic stages of *P*. *falciparum*, demonstrated by using transgenic parasites with a haemagglutinin-tagged version of FPPS. Also, the present data demonstrate that the recombinant protein is inhibited by risedronate.

**Conclusions:**

The rPfFPPS is a bifunctional FPPS/GGPPS enzyme and the structure of products FOH and GGOH were confirmed mass spectrometry. Plasmodial FPPS represents a potential target for the rational design of chemotherapeutic agents to treat malaria.

## Background

Malaria is a leading cause of morbidity and mortality in tropical regions. In 2010, there were an estimated 216 million episodes of malaria of which approximately 81%, or 174 million cases, occurred in the African continent. On a worldwide scale, 655,000 individuals died of malaria, most of them in sub-Saharan Africa [1]. Of the five parasite species that infect humans, *Plasmodium falciparum* is responsible for the vast majority of severe forms of, and deaths from, the disease. Recent observations alert that the parasite is becoming resistant to virtually all drugs currently used in the treatment of the disease. Efforts to tackle this problem are based on combined therapy using drugs to which the parasites have not yet developed resistance, as well as identifying new drug targets [2].

**Figure 1:**
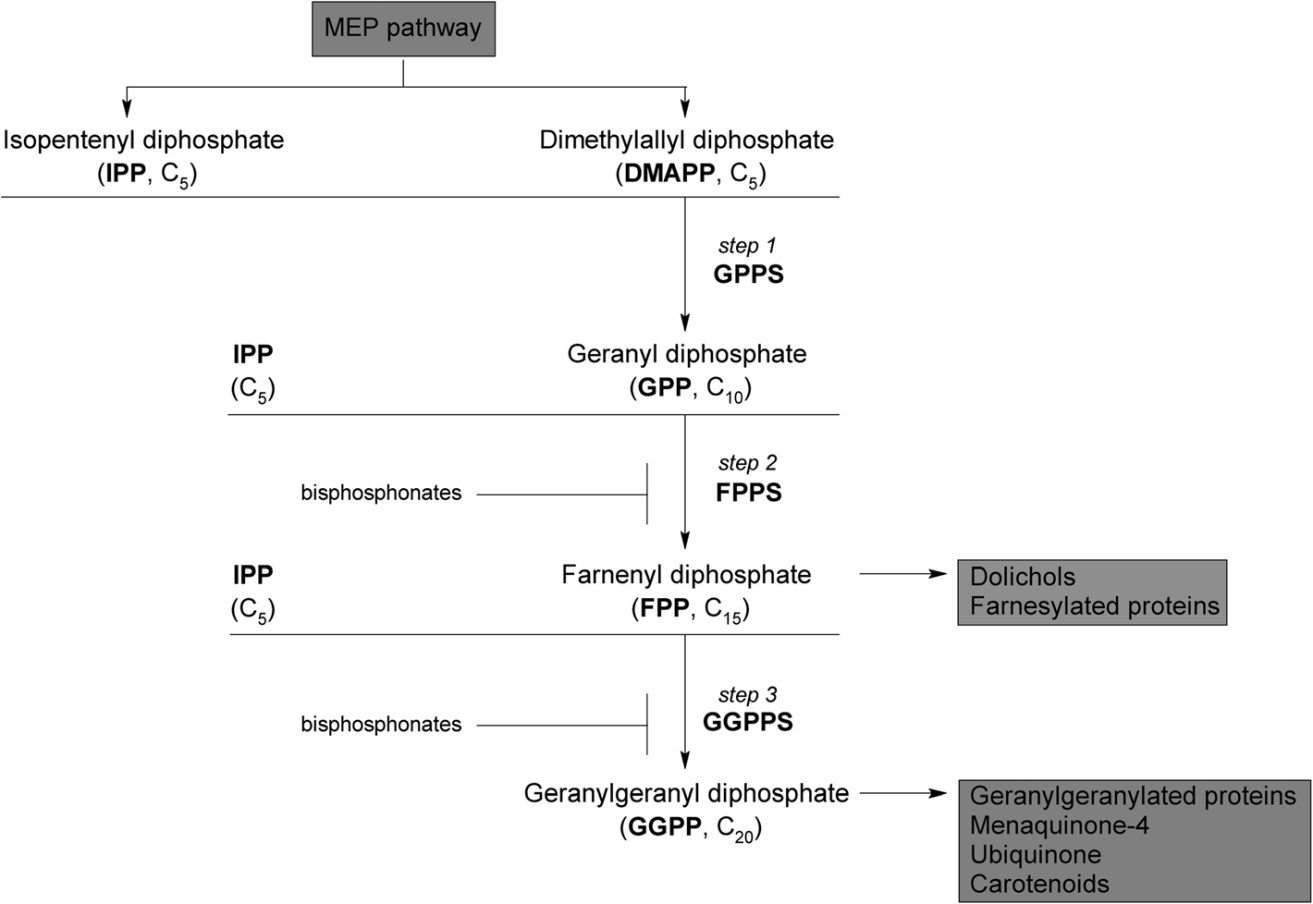
**Schematic diagram of the isoprenoid biosynthesis and downstream products in *Plasmodium falciparum*.** Bisphosphonates are known to inhibit FPPS/GGPPS, thereby preventing the synthesis of FPP and GGPP required for the biosynthesis of ubiquinone, dolichol, carotenoids, menaquinone, tocopherol, and protein prenylation. MEP: methyl erythritol phosphate.

*Plasmodium falciparum* parasites harbour an unusual plastid organelle called the apicoplast that has an essential role for their survival since isoprenoid precursors are synthesized there [3]. Deletion of this organelle by concomitant supplementation with isopentenyl diphosphate (IPP) proved that this is the only essential function of the apicoplast during blood stage growth [4]. Isoprenoids are very diverse and constitute an abundantly present group of natural products. Synthesis of isoprenoids is intrinsic to all organisms and leads to a vast array of metabolites with diverse functions. Despite their structural and functional variety, all isoprenoids derive from a common precursor, isopentenyl diphosphate, and its isomer, dimethylallyl diphosphate (DMAPP). Farnesyl diphosphate synthase (FPPS), which belongs to a family of enzymes classified as prenyltransferases, catalyzes the consecutive head-to-tail condensation of IPP with DMAPP to form geranyl diphosphate (GPP), and then a second condensation between GPP and IPP to form farnesyl diphosphate (FPP). FPP serves as a substrate for the first reaction of several branched pathways leading to the synthesis of compounds such as ubiquinone, dolichol, menaquinone, and prenylated proteins. FPP can also be condensed with an additional IPP by geranylgeranyl diphosphate synthase (GGPPS) to form geranylgeranyl diphosphate (GGPP), which is also employed in protein prenylation and is related to carotenoid biosynthesis (Figure 1).

The essential and major biosynthetic step in the metabolism of all isoprenoid is the elongation of isoprene units by prenyltransferases. These enzymes, which subsequently mediate alkylation of IPP by allylic diphosphate, are classified according to the chain length of the final product and stereochemistry of the double bond formed by condensations. FPPS and GGPPS are the most studied prenyltransferases and have been described in various organisms of all three kingdoms, Eukarya, Bacteria, and Archaea [5]. In protist parasites, the FPPS gene was cloned from *Trypanosoma cruzi*[6], *Trypanosoma brucei*[7] and *Toxoplasma gondii*[8]. Recently, a GGPPS from *Plasmodium vivax* was described [9]. However, the first characterization of a prenyltransferase in a malaria parasite was the characterization of the octaprenyl diphosphate synthase (OPPS) that catalyzes the condensation of FPP with IPP to produce octaprenyl diphosphate [10].

Human FPPS has been found to be a target for nitrogen-containing bisphosphonate (N-BP) drugs [11]. Based on “growth-rescue” and enzyme inhibition experiments, human GGPPS was shown to be a major target for the lipophilic analogues zolendronate and risedronate [12]. These reports have generated considerable interest in FPPS as a promising target for new anti-malarial drug development. Jordão *et al*. suggested the possible mechanism of action for risedronate in *P*. *falciparum* by inhibition of FPPS [13]. In the causative agent of sleeping sickness, *T*. *brucei*, the inhibition of FPPS showed that this enzyme is essential for parasite survival [7]. Considering that FPPS is a key enzyme of the biosynthesis of compounds already characterized in the parasite, such as dolichols, farnesylated proteins, and other final isoprenoid products [14], it is essential to characterize the FPPS from *P*. *falciparum* in order to establish an appropriate strategy for the development of specific inhibitors.

This work describes the cloning, expression and characterization of recombinant *P*. *falciparum* FPPS (rPfFPPS), with catalytic activity for DMAPP, GPP, and FPP as substrates, yielding FPP and GGPP as final products. Apparent kinetic parameters for the recombinant enzyme are presented, as well as IC_50_ and apparent *K*_*i*_ values for risedronate inhibition of rPfFPPS enzyme activity. Constitutive protein expression is also described.

## Methods

### *Plasmodium falciparum* culture

Cultures of *P*. *falciparum* clone 3D7 were grown as described [15], replacing human serum with Albumax I (0.5%, Invitrogen/Life Technologies) [16]. Parasite development and multiplication were monitored by microscopic evaluation of Giemsa-stained thin smears. Schizont stages were purified with magnetic columns (MACS Separation Columns “CS”, Miltenyi Biotec) [17]. Column pre-equilibration, washing and elution were all carried out at room temperature with RPMI-1640 (Sigma-Aldrich). For schizont purification, the culture was centrifuged (2,000 x *g* for 5 min), the pellet resuspended in RMPI-1640 (1:10; v/v), 10 ml of the 10% suspension of erythrocytes were applied to a CS column assembled in a magnetic unit, where only schizonts are retained. After washing the column with 50 ml of RMPI-1640, the column was removed from the magnetic field and its contents eluted with 50 ml of RMPI-1640 and the schizont stage parasites were centrifuged at 2,000 × *g* for 5 min at room temperature. The supernatant was discarded, and the pellet of parasites was stored in liquid N_2_ for subsequent analysis.

### Isolation and cloning of gene PF3D7_1128400

A 1,131 bp fragment of the PfFPPS gene (PlasmoDB ID PF3D7_1128400) was amplified from cDNA from mixed stage parasites using primers (Invitrogen/Life technologies) PfFPPS1 (5′-CC**GGATCC**ATGGAGAACGAGCAGAATAAC-3′) and PfFPPS2 (5′-CG**GAATTC**TCAAGCGCCTGTAAACAAAATGTC-3′) and the amplicon cloned in pGEM T easy (Promega). After sequencing, the fragment containing the complete ORF was subcloned in pGEX2T for recombinant protein expression using the introduced BamHI and EcoRI sites.

### Expression and Purification of rPfFPPS from *Escherichia coli*

Recombinant pGEX-2 T-FPPS expression vector was used to transform *Escherichia coli* BL21(DE3+) pLys RIL cells. Bacterial clones were grown in LB medium containing 50 μg/ml ampicillin and 34 μg/ml chloramphenicol at 37°C in Luria Broth (Hi-media) until an OD_600_ of 0.6. At this time point, the expression of rPfFPPS was induced with 0.2 μM isopropyl β-D-thiogalactoside at 24°C overnight. Cells were pelleted by centrifugation and resuspended in lysis buffer PBS/0.1% Triton X-100 pH 7.2 (v/v), 0.05 mg/ml lysozyme and 0.2 mM PMSF. Lysis was completed by sonication (five pulses of 30 s at 40 W, at 4°C). Recombinant proteins were then purified using glutathione sepharose beads (GE Healthcare), following the manufacturer’s instructions. Proteins were checked for purity by SDS-PAGE [18] and quantified by the Bradford method [19].

### Enzymatic activity assay

The catalytic activity of rPfFPPS was assayed by measuring the conversion of [4-^14^C]IPP (56.6 mCi/mmol, Perkin Elmer Life Sciences) to [^14^C] products, by two different protocols: **Protocol I** - The method described by Ling *et al*. [8] was used with some modifications. Briefly, the assay mixtures contained 10 mM HEPES buffer pH 7.4, 2 mM MgCl_2_, 2 mM dithiothreitol, 100 μM [4-^14^C]IPP, an allylic substrate (100 μM DMAPP, 30 μM GPP, or 15 μM FPP), and 500–1,000 ng of recombinant protein in a total volume of 100 μl. The reaction was carried out at 37°C for 30 min and stopped by addition of 10 μl of 6 M HCl. The reaction mixture was neutralized by addition of 15 μl of 6 M NaOH. The alcoholic products were then extracted twice with 500 μl hexane and analysed by reverse phase thin layer chromatography (RP-TLC). All non-radioactive substrates and chemicals were from Sigma-Aldrich. **Protocol II** - rPfFPPS activity was measured by a modification of the method described by Chang *et al*. [20]. Final assay concentrations were 50 mM Tris–HCl buffer pH 7.5, 2 mM MgCl_2_, 5 mM iodoacetamide, and 500–1,000 ng of recombinant protein. The concentrations of allylic substrate, DMAPP, GPP and FPP were the same as described above. The final reaction volume was 100 μl. After pre-incubation at 37°C for 10 min, the reaction was started by adding 50 μM [4-^14^C]IPP. The mixture was incubated at 37°C for 30 min and the reaction was terminated by addition of distilled H_2_O and NaCl-saturated water. The diphosphate products were then extracted twice with 500 μl of 1-butanol saturated with NaCl-saturated water and analysed by reverse phase high-performance liquid chromatography (RP- HPLC). Enzyme activity measurements using [1-(n)-^3^H]FPP (15 Ci/mmol, Amersham Biosciences) and IPP as substrates were also carried out.

### Identification of reaction products of rPfFPPS

The alcoholic products obtained by **Protocol I** were analysed by TLC on reverse phase Silica Gel 60 plates (Merck) with acetone:H_2_O (6:1; v/v) [8]. The position of the standard prenyl alcohol was visualized using iodine vapour. Radioactivity was visualized by autoradiography in a Storm phospho-imager. The diphosphorylated products that were formed following **Protocol II** were identified by RP-HPLC and analysed on a Phenomenex Luna C_18_ column (250 mm × 4.6 mm × 5 μm) (Phenomenex) coupled with a C_18_ pre-column (Phenomenex), a UV Gilson 152/UV variable UV/visible detector at 214 nm and an FC203B fraction collector. The software used for data processing was the UniPoint LC™ 3.0 Software System. The gradient elution system used was: solvent A, 25 mM NH_4_HCO_3_, pH 8.0; solvent B, 100% (v/v) acetonitrile. A linear gradient was run from 0% to 100% B over a period of 40 min, after which 100% B was then pumped through for an additional 5 min. Fractions were collected in 1 ml/min intervals [21]. The resulting fractions were dried, resuspended in 500 μl of liquid scintillation mixture (PerkinElmer Life Sciences) and monitored with a Beckman 5000 ***β***-radiation scintillation counter (Beckman).

### ESI-MS/MS investigation of the products geraniol (GOH), farnesol (FOH) and geranylgeraniol (GGOH)

Identification of product formation by using **Protocol I** with non-radioactive substrates (IPP/DMAPP) in the presence of rPfFPPS were carried out by electrospray ionization tandem mass spectrometry (ESI-MS/MS) using a ion trap mass spectrometer, model LCQ™ Duo (Thermo Scientific) coupled to a nano-HPLC system (Ultimate, Dionex). After stopping the reaction, products were extracted with hexane, dried in a vacuum centrifuge, and resuspended in 40 μl of 50% acetonitrile/0.2% formic acid. The sample was injected (10 μl) in the nano-probe of the spectrometer by an autosampler (Ultimate, Dionex) at a flow rate of 2 μl/min and analysed in the positive mode, using the following parameters: spray voltage 1.8 kV, capillary voltage 38 V, and capillary temperature 180°C. For ESI-MS/MS, relative collision energy of 30% (1.5 eV) was applied.

### Partial purification of native PfFPPS

The partial purification of native PfFPPS was performed only with schizont stage parasites purified by magnetic column separation, as described above. Partial protein purification was carried out according to Tonhosolo *et al*. [10]. **Protocol II** was used to assay the enzymatic reaction and the diphosphate products were analysed by RP-HPLC, as described above.

### rPfFPPS kinetic assays

For determination of apparent kinetic constants, concentration of the first substrate DMAPP (0–150 μM), GPP (0–50 μM) or FPP (0–50 μM) was varied in the presence of a fixed concentration of [4-^14^C]IPP (50 μM). Enzyme activity measurements were also carried out varying the concentration of [4-^14^C]IPP (0–80 μM) in the presence of a fixed concentration of either DMAPP (100 μM), GPP (40 μM) or FPP (50 μM). The catalytic activity of rPfFPPS was assayed by measuring the conversion of [4-^14^C]IPP to [^14^C] products, as described in **Protocol I**. Reaction products were extracted with hexane and quantified by liquid scintillation counting. Apparent kinetic constants, *K*_*M*_ and *V*_*max*_, for each substrate were derived from fitting the data to Michaelis-Menten (MM, Equation 1), using SigmaPlot 10, from Systat Software. All experiments were performed in triplicate.

(1) $$v=\frac{\mathrm{Vmax}\;\left[S\right]}{\mathrm{KM}+\left[S\right]}$$

### rPfFPPS inhibition assays

Inhibition assays were performed in presence of a fixed concentration (30 μM) of one allylic substrate (GPP or FPP) and fixed concentration (30 μM) of [4-^14^C]IPP, with varying concentrations of risedronate (0.005 – 1,000 μM). Each assay contained 500 ng of rPfFPPS in a final volume of 100 μl. The catalytic activity of rPfFPPS was measured by the conversion of [4-^14^C]IPP into [^14^C] products, as described in **Protocol I**

The concentration of risedronate required to reduce the fractional enzyme activity to half of its initial value in the absence of inhibitor (IC_50_) was obtained from fitting the data to Equation (2) for partial inhibition [22], in which *y* is the fractional activity of the enzyme in the presence of inhibitor at concentration [I]; *y*_(max)_ is the maximum value of *y* observed at [I] = 0; and *y*_min_ is the minimum limiting value of *y* at high inhibitor concentrations. Data analysis was performed using SigmaPlot 10 (Systat Software). Relationship between IC_50_ and risedronate apparent dissociation constant (*K*_*i*_) in each assay was derived according to Cheng’s and Prusoff’s relationship, Equation (3), for competitive inhibitors [23,24], in which [*S*] and *K*_*M*_ are, respectively, the concentration of the substrate for which risedronate is a competitive inhibitor, and this substrate MM constant. All experiments were performed in triplicate.

(2) $$y=\frac{y_{\max}-y_{\min}}{1+\frac{\left[I\right]}{IC_{50}}}+y_{\min}$$

(3) $$K_{i}=\frac{IC_{50}}{1+\frac{\left[S\right]}{K_{M}}}$$

### Plasmid construction

The plasmid pTEX150-HA/Stre3 [25] containing the epitope of heamagglutinin (HA) was digested with BglII/PstI to release the gene pTEX150. The genomic DNA sequence encoding the C-terminal fragment of FPPS was PCR amplified with the oligonucleotides 5′-AGATCTGGTATGCAAATGGGGGGTATA and 5′-CTGCAGCAGCGCCTGTAAACAAAATGTC, cloned in pGEM T-easy (Promega) and verified by sequencing. A recombinant clone was digested with BglII/PstI and ligated into the pTEX150 depleted vector pTEX150-HA/Stre3 generating the plasmid pFPPs-HA.

### Parasite transfection and characterization of transfectants

Parasites were transfected as previously described [26], using the electroporation conditions established elsewhere [27]. Briefly, *P*. *falciparum* 3D7 was cultured in 4**%** haematocrit in RPMI HEPES supplemented with 0.5% Albumax I. 2 × 10^7^ ring stage parasites at 5-8% parasitaemia were transfected with 150 μg of plasmid. Transfected parasites were submitted to drug pressure with 2.5 nM WR99210 starting on the third day of culture. Parasites were cultivated in standard conditions until parasites re-appeared and normal growth was re-established. The integration at the genomic FPPS locus was forced by intermittent exposure and retrieval of WR99210. Genomic gDNA was purified using standard protocols [28]. The integration at the genomic locus was checked by PCR under standard conditions using oligonucleotides inside and outside the integrated locus. The details of plasmid construction and the integration are presented on Additional file 1.

### Western blot analyses

Synchronous cultures of transfected *P*. *falciparum* were recovered in each stage. Ring, trophozoite or schizont stages were treated with 0.15% saponin in RPMI media to release haemoglobin from the red blood cells. Proteins were extracted with buffer: 0.05 M Tris–HCl, pH 6.8, 10% glycerol, 2 mM EDTA, 2% SDS, 0.05% bromophenol blue, 50 mM dithiothreitol [29] for separation by gel SDS-PAGE. The gel was then transferred to nitrocellulose membrane (Amersham) for 1 h using a Trans-Blot semidry electroblotter (BioRad) [30]. After blocking, membranes were incubated with an α-HA monoclonal antibody (1:500 dilution; Sigma-Aldrich) or antibody controls α-PTEX150 (1:1,000) [25] or α-MSP2 (1:500) [31] for 1 h at room temperature or 14 h at 4°C. After this, the membranes were incubated with an anti-mouse IgG labelled secondary antibody with peroxidase and were visualized on radiographic film using the ECL enhanced chemiluminescence detection kit according to the instructions of the manufacturer (GE Healthcare).

### Sequence analysis of the chain length determination region

For sequence selection, similarity searches were done using PfFPPS as query against the full NCBI nr protein database, using a maximum E-value cut-off of 10^-10^. Sequences with 0.5 to 1.5 times the length of the *P*. *falciparum*'s protein and those identical to others were removed. The final set of protein sequences was aligned using muscle 3.8.31 [32] and analysed with WebLogo 2.8.2 [33] and in-house developed scripts for amino acid composition analysis. Based on sequence conservation, 9 alignment columns either side of the First Aspartate-Rich Motif (FARM) were analysed and only sequences containing the canonical DDxxD motif were kept. Accession numbers and CLD region for all sequences used can be found in Additional file 2.

### Ethical statement

This study was approved by the Ethical Committee of the Institute of Biomedical Science of University of São Paulo,Brazil (CEUA 140.09).

## Results

### Expression and purification of recombinant protein

The *P*. *falciparum* gene PF3D7_1128400 was formerly annotated as an FPPS and is currently described as a GGPPS according to plasmoDB. Using this sequence as template, primers were designed to amplify PF3D7_1128400 from total cDNA by PCR. The full length protein was expressed as a GST fusion protein in *E. coli* BL21(DE3) pLys RIL cells and purified the protein by affinity chromatography as described in the Methods section. The protein homogeneity was inferred by SDS-PAGE followed by Coomassie Blue staining, showing that the purified GST-PfFPPS (rPfFPPS) protein has an apparent molecular mass of ~ 70 kDa, (sum of 26 kDa GST and 44 kDa PfFPPS) (Additional file 3).

### Catalytic activity of rPfFPPS

To verify if the PF3D7_1128400 gene encodes a functional rPfFPPS protein, its catalytic activity was assessed using the substrate [4-^14^C]IPP and three different allylic substrates DMAPP, GPP or FPP under the conditions described above. The reaction products were identified by TLC and RP-HPLC.

**Figure 2:**
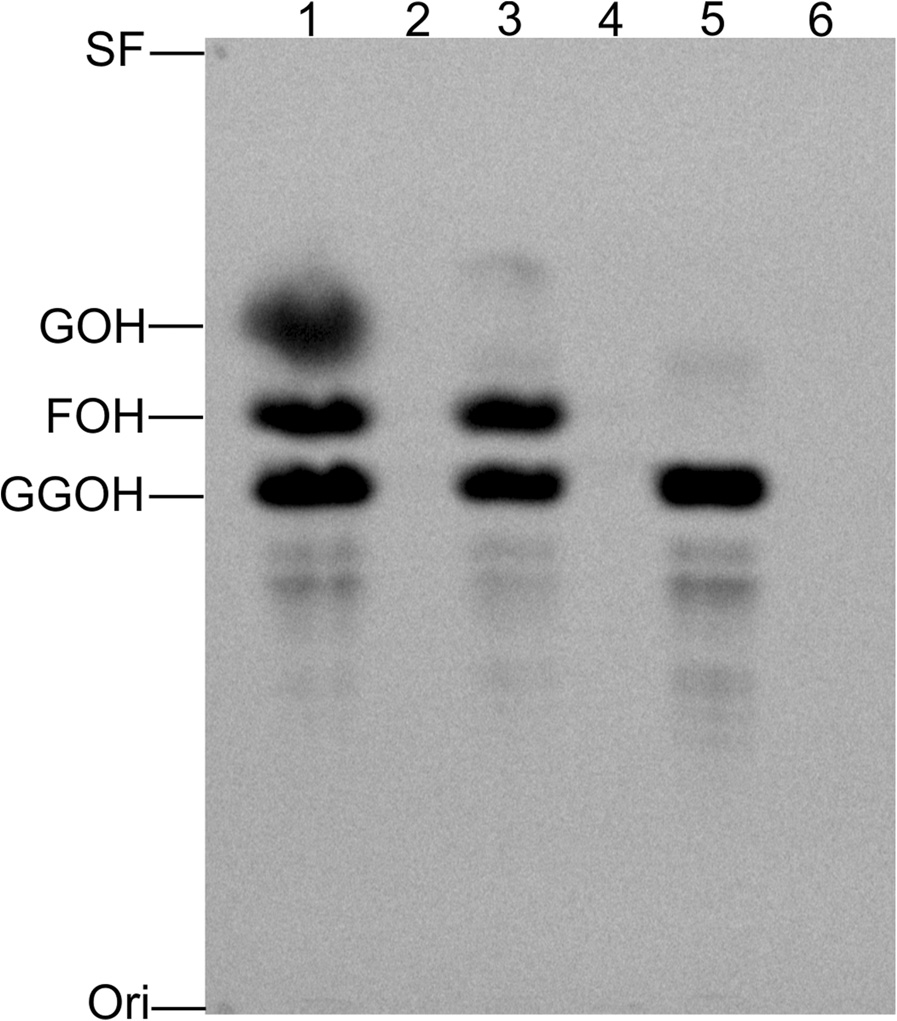
**Analyses by TLC of products synthesized by rPfFPPS.** The activity of rPfFPPS was measured by a ^14^C radioactivity assay, utilizing [4-^14^C]IPP and DMAPP, GPP, or FPP as allylic substrate. The enzymatic reactions (**Protocol I**) and TLC were performed as described in the Methods section. Lane 1, [4-^14^C]IPP + DMAPP as substrates; lane 3, [4-^14^C]IPP + GPP as substrates; lane 5, [4-^14^C]IPP + FPP as substrates; lanes 2, 4 and 6 control reactions without enzymes for reactions showed on lanes 1, 3 and 5 respectively. Products labelled with [4-^14^C]IPP were visualized by a Bioscan System 200 Imaging Scanner. The positions of geraniol (GOH), farnesol (FOH) and geranylgeraniol (GGOH) standards are indicated on the left. The main products detected were FOH and GGOH, indicating that this were the major enzyme products. Ori: origin SF: front.

The products formed following **Protocol I** were extracted with hexane, and the respective alcohols were submitted to TLC analysis. With the substrates [4-^14^C]IPP and DMAPP, bands with *R*_*f*_ values corresponding to GOH, FOH, and GGOH were observed. Bands with similar *R*_*f*_ to FOH and GGOH were detected when [4-^14^C]IPP and GPP were used as substrates, whereas FPP and [4-^14^C]IPP yielded only a band with an *R*_*f*_ coincident with GGOH (Figure 2). When the enzymatic reaction was carried without any enzyme, no products were observed (Figure 2, lanes 2, 4 and 6). When the enzymatic reaction was carried out with purified GST only, no products were observed.

**Figure 3:**
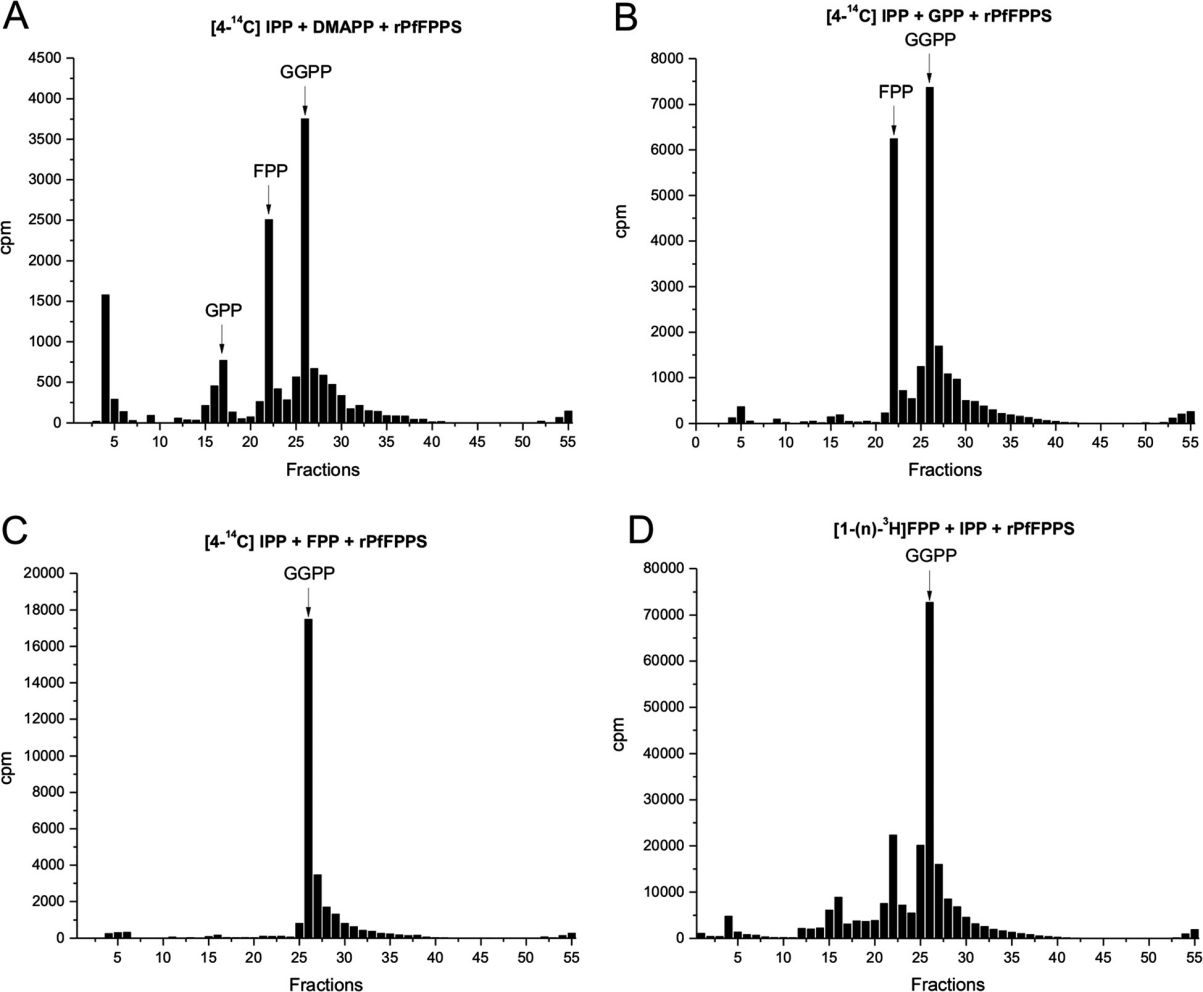
**RP-HPLC analysis of the product spectrum synthesized by rPfFPPS.** The enzymatic reactions (**Protocol II**) and RP-HPLC procedure were performed as described in the Methods section. A) [4-^14^C]IPP and DMAPP; B) [4-^14^C]IPP and GPP; C) [4-^14^C]IPP and FPP; D) [1-^3^H]FPP and IPP. Arrows indicate the co-elution positions of isoprenoid standards. The retention times of GPP, FPP and GGPP were identified by co-injection of commercial standards.

The diphosphorylated products formed following **Protocol II** were extracted with butanol-satured water and analysed by RP-HPLC. rPfFPPS with allylic substrates [4-^14^C]IPP and DMAPP was able to catalyze the synthesis of GPP, FPP, and GGPP (Figure 3A), whereas the reaction incubated with [4-^14^C]IPP and GPP as substrates led to the biosynthesis of FPP and GGPP (Figure 3B). When [4-^14^C]IPP and FPP were used as substrates, only GGPP synthesis was observed (Figure 3C). Similar results were obtained when the substrates [1-(n)-^3^H]FPP and IPP were incubated with the rPfFPPS (Figure 3D). When the reaction was carried without enzyme, no products were observed. This indicates that major products of the reactions catalyzed by the enzyme rPfFPPS are FPP and GGPP, with a minor production of GPP, showing both FPPS and GGPPS activity using two different protocols.

### Identification of rPfFPPs products by ESI-MS/MS

**Figure 4:**
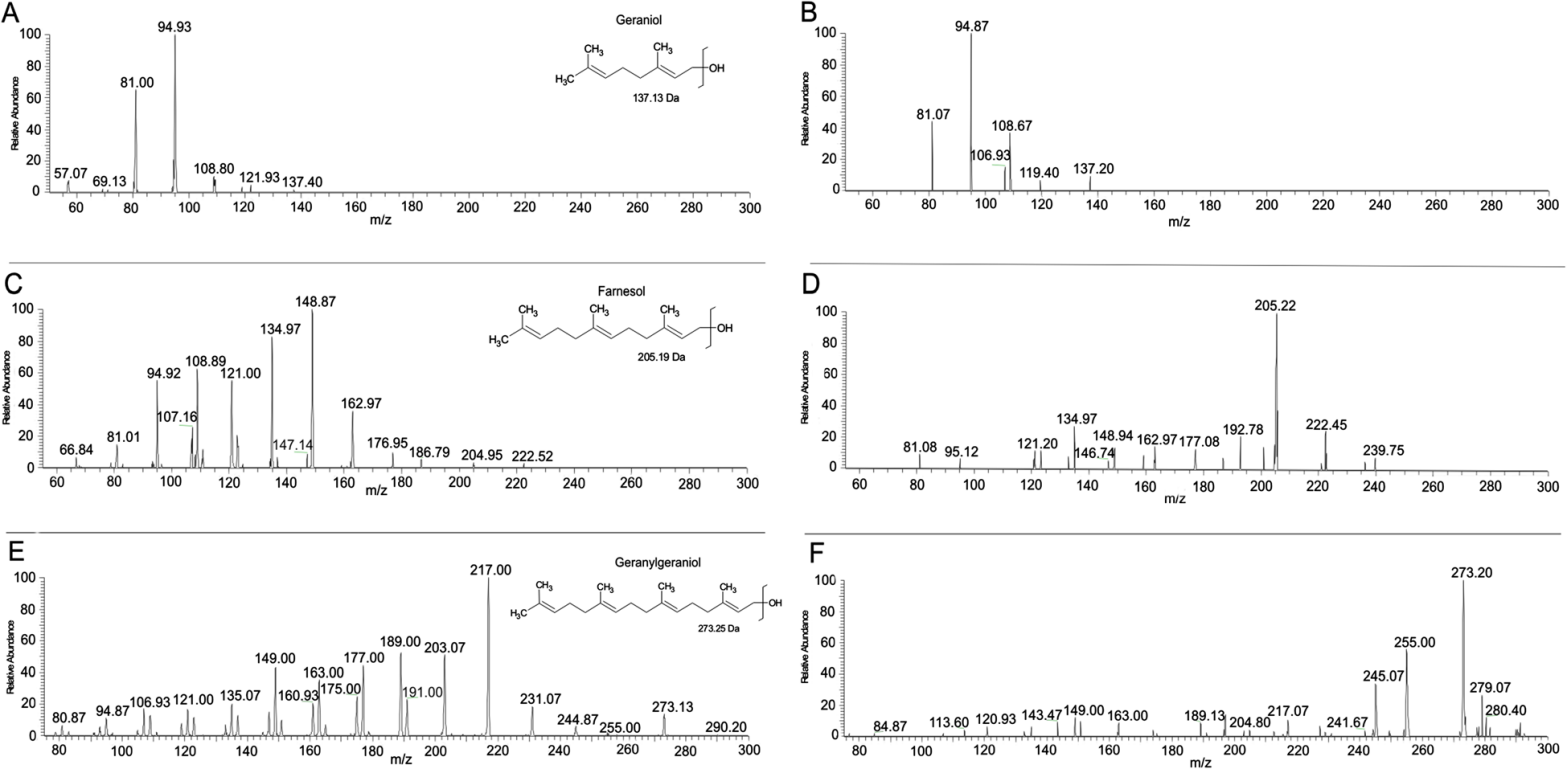
**ESI-MS/MS analysis of the products synthesized by rPfFPPS.** The *in vitro* enzymatic reaction was conducted as described in the Methods section with non-radioactive substrates. In plates A, C and E show fragmentation spectra of GOH at *m/z* 81, 94 and 108, FOH at *m/*z 121, 134, 148 and 162 and GGOH at *m/*z 163, 189 and 217 standards. Plates B, D, and F illustrates matching diagnostic dissociation profiles for the product of enzymatic reaction: GOH, FOH, GGOH.

In order to further confirm the product spectrum formed of rPfFPPS, isoprenic compounds were also identified by mass spectrometry. **Protocol I** was used for measurements of enzyme activity with non-radioactive substrates IPP/DMAPP, and investigated the structures of compounds formed in the presence of rPfFPPS by ESI-MS/MS (Figure 4). Figure 4A, C and E present the MS/MS spectra of standards GOH, FOH, and GGOH respectively. The fragmentation patterns of the precursor ions at *m/z* 137, corresponding to dehydration of GOH [M-H_2_O]^+^, at *m/*z 205; corresponding to the dehydration of FOH [M-H_2_O]^+^; and at m/z 273, corresponding to the dehydration of GGOH [M-H_2_O]^+^, were compared between standards and samples. The dissociation of the precursor ion at *m*/z 137 (GOH) revealed the presence of major ions at *m/z* 81, 94, and 108, while the dissociation of the precursor ion at *m*/z 205 (FOH) resulted in the major ion products at *m/z* 121, 134, 148, and 162. GGOH precursor ion at *m/z* 273 revealed the product major ions at *m/z* 149, 163, 189 and 217. The molecular identity was confirmed by comparing the ESI-MS/MS spectrum of the ions at m/z 137, *m/*z 205, and *m/*z 273 produced by rPfFPPS (Figure 4B, D and F) with the ESI-MS/MS spectrum of the standards (Figure 4A, C and E), revealing the same dissociation profile. Taken together, these results underscore that the rPfFPPS is able to catalyze reactions that lead to GOH, FOH, and GGOH formation.

### Characterization of PfFPPS activity in parasite extracts by HPLC

**Figure 5:**
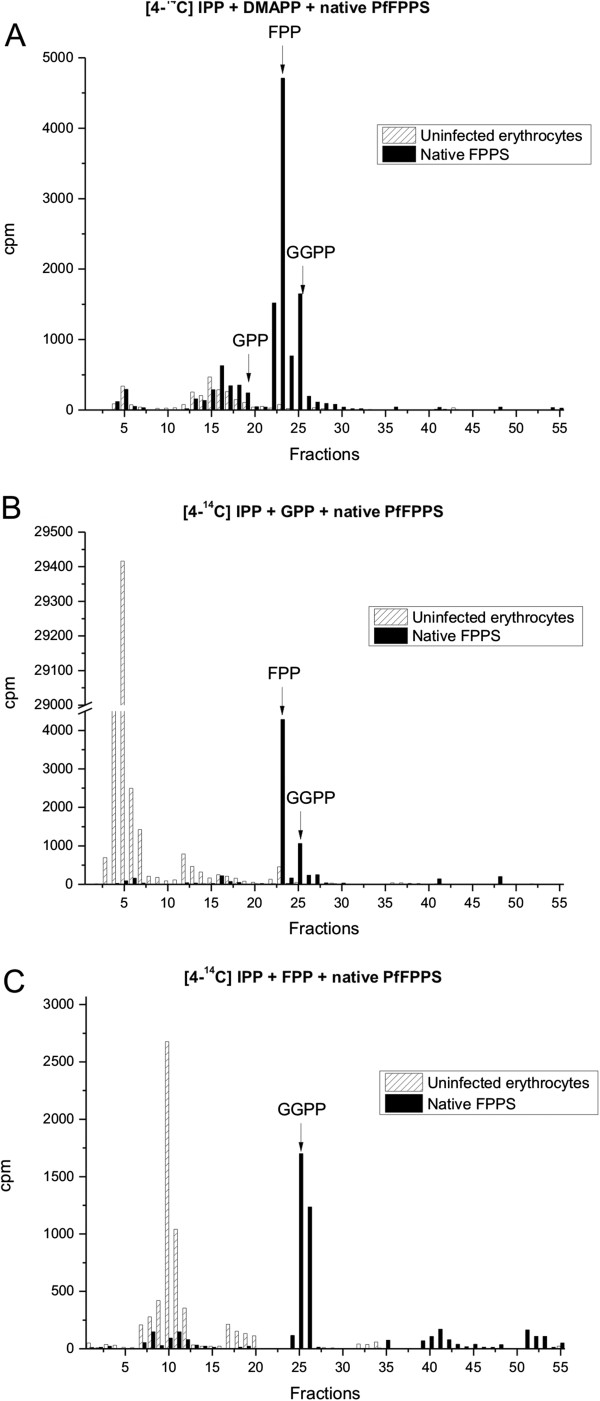
**RP-HPLC analysis of radiolabeled products biosynthesized by the native PfFPPS in 5 × 10**^**10 **^**semipurified *Plasmodium falciparum *schizont stages.** Extracts of parasites were obtained with methodology described by Tonhosolo *et al*. [10]. The reaction of [4-^14^C]IPP with three allylic substrates: DMAPP, GPP, or FPP were realized. Arrows indicate the elution positions of isoprenoid standards. The retention times of GPP, FPP and GGPP were identified by co-injection of commercial standards. (**A**) DMPP substrate, (**B**) GPP substrate, and (**C**) FPP substrate.

In order to verify if naturally occurring PfFPPS contained in *P*. *falciparum* extracts exert similar activities as detected with rPfFPPS, these extracts were used instead of recombinant protein. The reaction was performed with [4-^14^C]IPP and DMAPP, GPP or FPP as substrate in accordance with **Protocol II**. The products were analysed by RP-HPLC. Incubation of extracts in the presence of [4-^14^C]IPP and DMAPP led to formation of GPP, FPP, and GGPP. Likewise, incubation of [4-^14^C]IPP andGPP as substrates yielded FPP and GGPP as products.Finally, only GGPP was observed when extracts were incubated with [4-^14^C]IPP and FPP (Figure 5). The extracts of parasites exhibited both FPPS and GGPPS activity and these activities were similar to those of the rPfFPPS protein.

### Apparent kinetic parameters of rPfFPPS and IC_50_ of risedronate

Apparent kinetic constants of the recombinant enzyme were determined using varied concentrations of [4-^14^C]IPP, DMAPP, GPP, and FPP as substrates (Additional file 4). The parameters were determined as described in the Methods section, by measuring the radioactivity in the hexane fraction. *K*_*M*_ and *V*_*max*_ values for each substrate are given in Table 1. Risedronate inhibitory activity against rPfFPS, by specifically inhibiting the condensation of IPP with an allylic substrate was assayed as described in the Methods section. Risedronate inhibition was evaluated using FPP/IPP and GPP/IPP as substrates (Additional file 5), yielding, respectively, IC_50_ values of 1.3 ± 0.2 μM and of 10 ± 1 μM. Apparent *K*_*i*_ values, assuming risedronate competitive inhibition towards FPP and GPP, are equal to 0.08 μM and 1.96 μM respectively.

**Table 1:** **Apparent kinetic constants for *Plasmodium falciparum *FPPS**,(**rPfFPPS**)

**Varied substrate**	**Fixed substrate**	***K***_***M ***_**(μM)**	***V***_***max ***_**(nmol/min/mg)**
DMAPP (0–150 μM)	IPP 50 μM	68 ± 5	452.5 ± 16
GPP (0–50 μM)	IPP 50 μM	7.8 ± 1.3	341 ± 19
**FPP (0–50 μM)**	**IPP 50 μM**	**2.06 ± 0.4**	**326.5 ± 16**
IPP (0–80 μM)	DMAPP 100 μM	2 ± 0.3	169 ± 5.4
IPP (0–80 μM)	GPP 40 μM	0.81 ± 0.1	224 ± 3.4
**IPP (0–50 μM)**	**FPP 50 μM**	**2.4 ± 0.3**	**155.6 ± 4**

Concentration ranges for each varied substrate are indicated. Activity *versus* varied substrate concentration plots are depicted on Additional file 4.

### Analysis of rPfFPPS expression during the intra-erythrocytic cycle by Western blot

**Figure 6:**
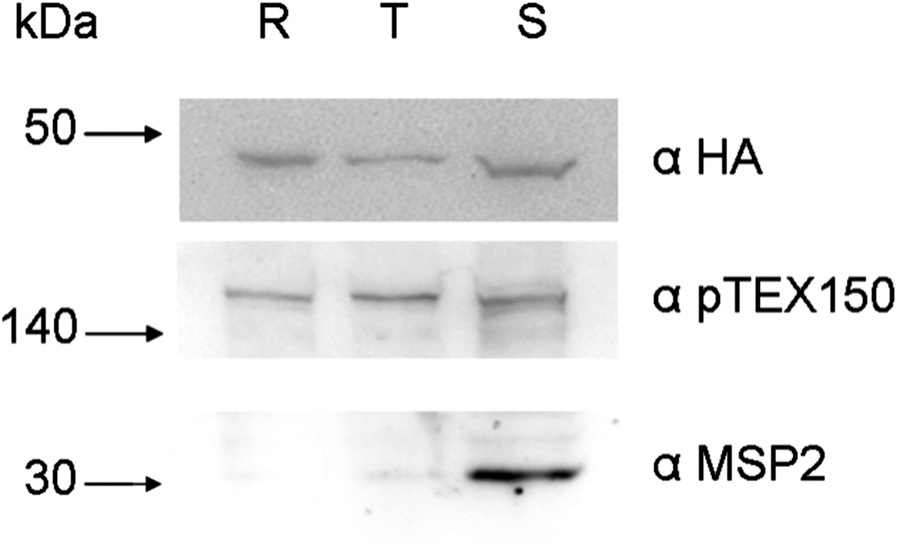
**Expression of FPPS protein during the three stages of the intra-erythrocytic cycle of *Plasmodium falciparum *using parasites transfected.** α-HA (antibody against the epitope of haemagglutinin protein (HA), encoded fused to the protein of interest-FPPS). Controls for the synchronization of intra-erythrocytic stages of the parasite: α-pTEX150 (antibody against pTEX150, protein constitutively present during the intra-erythrocytic cycle of the parasite), α MSP2 (antibody against MSP2, protein present only in the schizont stage). (R) ring trophozoite (T) trophozoite, (S) schizont. Molecular size standards are indicated on the left (kDa).

Extracts of parasite line that had the FPPS/GGPPS enzyme tagged with the HA epitope were analysed for the presence of pFPPs-HA. Samples of protein were extracted from parasites synchronized in three main stages (ring, trophozoite, and schizont) and detected with a monoclonal antibody against HA. The results indicate that the enzyme FPPS is constitutively expressed in all stages during the asexual intra-erythrocytic cycle of *P*. *falciparum* (Figure 6). As a control of the parasite synchronization, antibodies that recognize the constitutively expressed protein pTEX150 in three stages [25], and MSP2 [31], which is expressed only in schizont stages, were used.

### CLD region sequence analysis

The CLD regions of 452 sequences containing the canonical DDxxD FARM motif were analysed by creating a sequence logo showing relative amino acid frequencies (Additional files 2 and 6). There is clear predominance of aromatic amino acids (F and Y, although never W) in positions 4 and 5 N-terminal to the FARM (henceforth called P4 and P5). The cysteine in P5 with F or Y in P4, as found in *Toxoplasma*'s bifunctional FPPS/GGPPS, is very rare, occurring in only 6 sequences (1.33% of the total), of various taxonomic affiliations. The *P*. *falciparum* sequence (S in P5, aromatic in P4) is slightly more common, appearing in 14 sequences (3.10%). *Theileria* spp. and all plasmodia but *P*. *vivax* contain SF at those positions; other organisms of diverse taxonomic lineages present this same sequence arrangement. In contrast, the other two biochemically characterized bifunctional FPPS/GGPPS enzymes present FF (*Zea mays*) or FS (*M. thermoautotrophicum*) at these positions, with the former found in 174 (65.71%) of all sequences and the later present in only 22 (4.87%). Other positions in the sequence logo have also shown high levels of conservation (Additional file 2), most markedly positions 2–4 (most frequently LQA), 7–8 (LV), 12–13 (IM), 16 (S), 18–21 (TRRG), and 23 (P).

## Discussion

FPPS is a key enzyme in the metabolism of virtually all isoprenoids and it interconnects the 5-carbon moiety isoprenoid synthesis with the mid- or long-chained compound synthesis (Figure 1). In this study, the gene PfFPPS as encoding a bifunctional FPPS/GGPPS enzyme and its *in vitro* inhibition by risedronate were characterized.

In many organisms, the prenyltransferases that catalyze chain elongation are highly selective for the chain length of their products. The human genome contains genes for two distinct monofunctional enzymes for GGPP and FPP synthesis [34,35]. In the protozoans *T. cruzi* and *P. vivax*, either FPPS or GGPPS is present, respectively [6,9]. On the other hand, Artz *et al.* discuss the possibility that GGPPS of *P. vivax* could be a bifunctional enzyme [9].

rPfFPPS expressed as a GST-fusion protein was used to characterize its functional activity and to determine the apparent kinetic parameters. Interestingly, the removal of the GST tag from rPfFPPS resulted in almost complete activity loss. An active form of GGPPS from *Thermus thermophilus* and *Sulfolobus acidocaldarius* was also overexpressed in *E. coli* cells as a GST fusion protein. Ohto *et al.* suggested that the presence of the GST-tag leads to thermal stability of the recombinant enzymes [36].

Previous studies have shown that many FPPS homologues can accept both DMAPP and GPP as allylic substrates [37,38]. When synthesizing FPP from DMAPP, the enzyme catalyzes two condensation reactions with IPP, releasing only trace amounts of the intermediate GPP [39], while GGPPS can accept DMAPP, GPP, and FPP as substrates [40,41]. The activity of rPfFPPS and the parasite extracts were confirmed by purification of the synthesized products by RP-HPLC. When DMAPP was used as a substrate, GPP was detected in minor amounts while FPP and GGPP were the predominant products. When the reaction was catalyzed with GPP as allylic substrate, the only products observed were FPP and GGPP. Accordingly, when FPP was used as substrate, only GGPP was observed (Figure 3). No products were detected when GGPP was used as a substrate. Hence, rPfFPPS is a bifunctional FPPS/GGPPS enzyme. Importantly, similar products were observed using a second approach where alcoholic compounds were analysed by TLC (Figure 2). Finally, the structures of products GOH, FOH, and GGOH were confirmed by ESI-MS/MS (Figure 4).

The bifunctional property of rPfFPPS in producing GGPP as well as FPP was previously described only in three organisms: the archaebacterium *M. thermoautotrophicum*[42], maize [43], and *T. gondii*[8]. A related enzyme was described by Artz *et al.* in *Cryptosporidium parvum.* Although this enzyme was annotated as an FPPS, it shows the capacity to produce GGPP and also longer polyisoprenes (up to 35 carbons) with the main products being C25 and C30-compounds with most of the substrates tested [44]. This is indicative that the enzymes from *P. falciparum* and *T. gondii* have a rather limited product spectrum compared to the *Cryptosporidium* homologue.

Amino acid sequence alignment of FPPS from different organisms revealed conserved regions I to VII with two characteristic aspartate rich motifs, one in region II called FARM (first Asp-rich motif) and in region VI called SARM (second Asp-rich motif). Wang and Ohnuma [45] clearly demonstrated that the product chain lengths of natural FPPS and GGPPS are mainly regulated by the amino acid residues located at the fourth and fifth position upstream of the FARM region. These residues are at the bottom of the active site pocket, making direct interactions with the ***ω***-terminal region of the allylic products. For this reason, the site was designated the CLD (chain length determination) region. Usually, three possible amino acid substitutions are described for the fourth and fifth amino acid positions upstream the FARM region, and their identities determine the classification of FPPS and GGPPS: Type I FPPS present aromatic amino acids residues on both positions; Type II and Type III GGPPS present amino acid residues other than aromatic on both positions; Type II FPPS and Type I GGPPS, as well as long chain prenyl synthases, present an aromatic amino acid residue solely at the fifth position. Upon alignment of FPPS/GGPPS from *T. gondii* and GGPPS from *P. vivax* it appears that these proteins share more features with other FPPS as already postulated by Ling *et al.*[8], and FPPS from *P. falciparum* also falls in this cluster. Accordingly, these enzymes show the apparent production of GGPP and FPP, although this is not explicitly expressed in the characterization of the *P. vivax* enzyme [9]. One may argue that a hydrophilic side chain at the fifth amino acid upstream of the FARM region plays a crucial role for the production of both GGPP and FPP. Li *et al.*[46] showed that the presence of a cysteine at the fifth position is essential for the FPPS/GGPPS bifunctionality in *T. gondii*. On the other hand, the methanobacterial version of the enzyme contains a bulky phenylalanine at this position and also produces GGPP and FPP [42], turning evident that other regions may play a role in the fine-specificity of product formation. Our analyses of the CLD from 452 putative FPPS sequences show relatively high sequence conservation of other amino acids close to the FARM, and suggest the potential for the further discovery of a number of FPPS/GGPPS bifunctionality in organisms as diverse as animals, fungi, amoeba, plants, and others. From the point of view of parasitism, it is reasonable to infer that a bifunctional enzyme would be a selective advantage. Considering the notoriously reduced genomes in parasitic organisms and the fact that no other enzyme with similarity to known short chain prenyl synthases has been identified in the currently sequenced Apicomplexa, this mutation has probably been advantageous to these parasites given the essential nature of both FPP and GGPP as precursors of a number of compounds important for many processes of their cellular metabolism.

The results showed in TLC, HPLC, and ESI-MS/MS (Figures 2, 3 and 4) are indicative of bifunctional activity for rPfFPPS, showing catalytic activity with DMAPP, GPP, and FPP as first substrates, ultimately yielding GGPP as final product. Based on the conservation among FPPS and GGPPS enzymes, it is tempting to suggest that rPfFPPS mechanism of catalysis is bi-bi ordered, in which binding of either DMAPP, GPP, or FPP to the free enzyme is followed by IPP binding. However, other sequential or random mechanisms cannot be ruled out for the *P. falciparum* enzyme since the results here presented do not allow the determination of its kinetic mechanism. A mandatory ordered kinetic mechanism has been described for other FPPS, including the human [47], *T. cruzi*[48], *Staphylococcus aureus* and *E. coli*[49] homologues. According to such an ordered mechanism, DMAPP or GPP binds to the free enzyme, with IPP having larger binding affinity for the E:DMAPP or E:GPP binary complexes [47]. Farnesyl synthesis by these FPPS homologues is known to proceed through two subsequent steps. The reaction starts with the condensation of one molecule of DMAPP and one molecule of IPP, yielding the first product GPP. A second IPP molecule is condensed with GPP to form FPP as the final product [50]. Accordingly, *P. falciparum* bifunctional FPPS/GGPPS catalysis is a three-step, four-substrate process (Figure 1).

Data derived from activity assays of rPfFPPS were apparently hyperbolic to all tested substrate pairs (Additional file 4), suggesting that rPfFPPS follows MM kinetics. As rPfFPPS catalyzes parallel and consecutive reactions (Figure 1), the interpretation of the apparent kinetic constants for this complex enzyme system is not trivial (Table 1). The results presented here demonstrate that rPfFPPS is capable of synthesizing GPP, FPP, and GGPP from DMAPP and IPP (Figure 1, steps 1, 2 and 3); FPP and GGPP from GPP and IPP (Figure 1, steps 2 and 3); and GGPP from FPP and IPP (Figure 1, step 3). Assuming that rPfFPPS follows an ordered mechanism for substrate binding, when activity assays where carried out in the presence of DMAPP and IPP, there will be formation of GPP, followed by conversion of GPP to form FPP, which will be competitive inhibitors of the reactions catalyzed in steps 1, 2, and 3, since DMAPP, GPP, and FPP all compete for binding to the free enzyme active site (Additional file 7). On the other hand, rPfFPPS activity measurements using GPP and IPP as substrates, there will be formation of FPP, which will be competitive inhibitors of the reactions catalyzed in steps 2 and 3, since GPP and FPP compete for binding to free enzyme. In this scenario, DMAPP, GPP, and FPP will also behave as non-competitive inhibitors towards the second substrate, IPP (Figure 1, steps 1, 2 and 3). This same issue has been described for human FPPS [47], where the authors clearly point out the difficulties of mechanistic studies modelling and interpretation.

Evaluation of the apparent kinetic constants given in Table 1 should thus be interpreted with caution. Except for the substrate pair FPP/IPP (highlighted in bold), the parameters presented for every other pair of substrates correspond to overall dissociation constants (*K*_*M*_) and overall *V*_*max*_ values comprising the consecutive and parallel reactions that would be better described by modifications of the MM equation.

Similar *K*_*M*_ values for substrate pair IPP/FPP were reported for *Homo sapiens* GGPPS (3 ± 0.2 μM and 4.2 ± 0.3 μM) [34] and *P. vivax* GGPPS (8.4 ± 1.6 μM and 7.3 ± 0.7 μM) [9]. The *P. falciparum* substrate pair IPP/FPP also presented similar *K*_*M*_ values, of 2.4 ± 0.3 μM and 2.06 ± 0.4 μM (Table 1). The human FPPS enzyme has also been characterized, and *K*_*M*_ values for IPP/GPP of 0.6 ± 0.1 μM and 0.7 ± 0.1 μM were reported [47]. Again *P. falciparum* data for substrate pair IPP/GPP indicate similar *K*_*M*_ for IPP (0.81 ± 0.1 μM) and almost ten times larger *K*_*M*_ value for GPP (7.8 ± 1.3 μM). These values, however, correspond to global apparent constants for steps 2 and 3 (Figure 1).

Considering varied substrates DMAPP, GPP, and FPP, there appears to be a trend in MM constant values: *K*_*M*_(FPP) < *K*_*M*_(GPP) < *K*_*M*_(DMAPP) (Table 1). Increased *K*_*M*_ values, without *V*_*max*_ variations, are expected for reactions catalyzed in the presence of competitive inhibitors [22], as is the case for these substrates. No such *K*_*M*_ variation is expected when IPP is the varied substrate as IPP is a non-competitive inhibitor with respect to FPP, GPP, and DMAPP. Non-competitive inhibitors are expected to maintain *K*_*M*_ values while decreasing *V*_*max*_ values [22]. These predictions appear to be borne out by the data presented in Table 1.

Nitrogen-containing bisphosphonates like risedronate are known to inhibit FPPS enzymes [11]. However, when the activities of 26 different bisphosphonates against the GGPPS protein from *P. vivax* were compared to their effect on *P. falciparum in vitro* growth, a poor correlation was found [51]. Risedronate is commonly used in the treatment of osteoporosis and it was shown that risedronate has a significant inhibitory effect against murine blood stage malaria [13], also inhibiting *P. vivax* GGPPS [9], and human FPPS [47]. Jordão *et al.* showed that risedronate presents inhibitory activity *in vitro* cultures of *P. falciparum*, with an IC_50_ of 20 ± 1 μM, also showed that risedronate inhibition is reversed by addition of FPP or GGPP to the cultures, but not by the addition of IPP [13]. These findings are in agreement with the assumed competitive risedronate inhibition towards FPP and GPP, and non-competitive inhibition with respect to IPP.

As for the apparent kinetic constants reported in Table 1, an IC_50_ value of 10 ± 1 μM for risedronate inhibition in the presence of GPP/IPP substrates also corresponds to a global inhibition value, in which both risedronate and FPP product could account for the inhibitory activity. When risedronate effect was evaluated in the presence of FPP/IPP as substrates, an IC_50_ value of 1.3 ± 0.3 μM was estimated. The increased IC_50_ for the rPfFPPS/GGPPS reaction catalyzed with GPP/IPP as substrates is in agreement with the presence of an alternative substrate (FPP) as a competitive inhibitor [22]. A similar IC_50_ value was reported for the inhibition of human FPPS activity by risedronate. When GPP/IPP were used as substrates for FPPS enzyme activity measurements, in which there is no alternative substrate present in the reaction mixture, an IC_50_ value of 2.7 nM was determined. On the other hand, when DMAPP/IPP were the substrates, and reaction product GPP will also inhibit the enzyme along with risedronate, the IC_50_ value increased to 3.2 nM [47]. The larger IC_50_ values of risedronate in the presence of alternative substrates can be a consequence of some of the enzyme active sites being occupied by these substrates thereby increasing the concentration of inhibitor to achieve 50% of enzyme activity inhibition. In addition, *in vitro* inhibition assays of human FPPS also indicate that risedronate is a time dependent slow tight-binding inhibitor, with lower IC_50_ values after incubation for 30 minutes of enzyme in the presence of risedronate [47]. As described in the Methods section, rPfFPPS formation of products was evaluated only after 30 min incubation time, according to *Protocol I*. This thus prevents time dependent fluctuation of the IC_50_ value for the results presented here. Nonetheless, an alternative assay may be necessary to evaluate a possible tight-binding inhibition mechanism for risedronate over rPfFPPS.

With evidence of risedronate being a competitive inhibitor towards GPP and FPP, its apparent *K*_*i*_ value was estimated, according to Equation (3), as being equal to 1.96 μM (GPP/IPP) and 0.082 μM (FPP/IPP). *Plasmodium vivax* GGPPS characterization studies reported an apparent *K*_*i*_ value of 12.4 ± 1.7 μM, when using FPP/IPP as substrates [9]; a value 151 times larger than the *K*_*i*_ value reported in this work. Even though true *K*_*i*_ values must be assigned before a more reliable comparison can be made, *P. falciparum* FPPS/GGPPS seems to be more prone to risedronate inhibition than its *P. vivax* homologue. Reasoning for this finding is rather elusive at the moment.

Gosh *et al.* have shown that risedronate or zoledronate were not the most potent inhibitors in *Plasmodium* spp [52]. They recently described a new generation of bisphosphonates known as “liphophilic biphosphonates”, found to be more active against FPPS/GGPPS both *in vitro* and *in vivo* than any other currently available bisphosphonate [12]. In addition, No *et al.* demonstrated that the lipophilic analogues of risedronate and zolendronate had a stronger inhibitory activity against GGPPS from *P. vivax* and also exhibited anti-malarial activity *in vitro* and *in vivo*[53]. Although risedronate is not a potent drug against *P. falciparum*, it was showed by metabolic incorporation with [4-^14^C]IPP that risedronate inhibits the biosynthesis of FPP and GGPP and interferes with protein isoprenylation by inhibiting the biosynthesis of FPP and GGPP, while also interferes with the transfer of FPP to parasite proteins [13]. These findings are in agreement with the view that risedronate inhibits *in vitro P. falciparum* growth by inhibiting the plasmodial FPPS. Importantly, it is expected that successful inhibition of FPPS – a key enzyme between IPP/DMAPP and all longer polyisoprenoids – exerts a pleiotrophic effect on *Plasmodium* since it inhibits the function of many important parasite proteins [10,54].

The rPfFPPS is expressed constitutively in all stages during intra-erythrocytic cycle, demonstrated by using transfected parasites with pFPPS-HA (Figure 6). FPP and GGPP are substrates for prenyl:protein transferases (farnesyl transferase and geranylgeranyl transferase), catalyzing the post-translational modification of proteins [55]. Previous studies have demonstrated that post-translational modification of proteins occurs in all intra-erythrocytic stage of *P. falciparum*, suggesting that the enzyme is also active in all stages [55,56].

## Conclusions

The rPfFPPS is a bifunctional enzyme, with FPPS/GGPPS activity, producing FPP and GGPP. Both FPP and GGPP occupy a central role leading to the synthesis of important classes of compounds. These two compounds were utilized for demonstrating the several isoprenoid biosynthesis pathway in *P. falciparum*[14]. Considering that: i) *P. falciparum* does not survive in the absence of the IPP produced in the apicoplast unless this precursor is supplemented [4]; ii) the FPPS/GGPPS is the only enzyme leading to the precursors for the synthesis of larger polyisoprenoids; and, iii) that FPPS/GGPPS has major structural differences compared to the human FPPS and GGPPS enzymes [43], this enzyme possibly represents an attractive drug target for the development of selective inhibitors aiming the erythrocytic stages of *P. falciparum*. The results presented here and previously published data [13] on risedronate inhibition *in vitro* and *in vivo* call for further QSAR experiments for the development of more potent bisphosphonate-based inhibitors selectivity targeting this key point of the plasmodial isoprenoid metabolism.

## Abbreviations

IPP: Isopentenyl diphosphate; DMAPP: Dimethylallyl diphosphate; GPP: Geranyl diphosphate; FPP: Farnesyl diphosphate; GGPP: Geranylgeranyl diphosphate; FPPS: Farnesyl diphosphate synthase; GGPPS: Geranylgeranyl diphosphate synthase; N-BP: Nitrogen-containing bisphosphonate; OPPS: Octaprenyl diphosphate synthase; rPfFPPS: *P*. *falciparum* FPPS; ESI-MS/MS: Electrospray ionization tandem mass spectrometry; QSAR: Quantitative structure–activity relationship models.

## Competing interests

The authors declare that they have no competing interests.

## Authors’ contributions

FMJ initiated this work and performed most of the experiments, including Figures 1, 2, 3, 4 and 5, Additional files 3 and 5 and wrote the majority of the paper; HBG and MFdA performed the experiments in Figures 6 and Additional file 1; JMPA performed computational studies (Additional files 2 and 6); CBA performed ESI-MS/MS analyses; TDB, AB and LAB contributed in enzymatic kinetic analyses and wrote these aspects of paper. GW, EAK and AMK supervised the project, analysed the data, wrote and reviewed the paper before submission. All authors read and approved the final manuscript.

## Supplementary Material

Additional file 1 **Schematic representation of the integration of rPfFPPs-HA in genomic locus. A**) Diagram illustrating the integration event by crossing-over and primers designed to detect this event (1, 2 and 3). Numbers 1 and 3 indicate the region where the primers have been designed for detecting the integration of the gene in locus. **B**) PCR detecting the integration of pFPP-HA in the genomic locus of *P. falciparum* using primers 1 and 3. **C**) Detecting the control PCR amplification of endogenous FPPS gene in both strains (transfected and 3D7) using the primers 1 and 2. (−) –negative control; (**pFPPs**-**HA**) – transfected strain; (**3D7**) – wild type strain. Click here for file

Additional file 2 **Table of organisms, accession numbers, and CLD region sequences analyzed.** *Sequences characterized as bifunctional FPP/GPPS are highlighted in gray and use bold font, *Excluded from CLD analysis (X in red) were the sequences that either did not present the canonical DDxxD FARM motif or had rare insertions (see main text). Click here for file

Additional file 3 **Expression of the rPfFPPS. SDS-polyacrylamide gel 12% was stained with Coomassie Brilliant Blue.** Lane 1, soluble fraction from extract of *E. coli* BL21(DE3) pLys RIL/rPfFFPS; Lane 2, rPfFPPS fused with GST; lane 3, GST. Molecular size standards are indicated on the left (kDa). Click here for file

Additional file 4 **MM plots of the steady-state initial velocity experiments for rPfFPPS/GGPPS.** R2 values for each plot are: **A**) 0.99; **B**) 0.99; **C**) 0.98; **D**) 0.99; **E**) 0.97; **F**) 0.99. Experiments and data analysis were conducted as detailed under **Methods**, *rPfFPPS kinetic assays* section. Concentration ranges of each varied substrate are depicted on Table 1. Data were fitted to Equation (1). Click here for file

Additional file 5 **Inhibition of rPfFPPS/GGPPS activity by risedronate. A**) Substrate pair FPP/IPP (R2 = 0.98); **B**) Substrate pair GPP/IPP (R2 = 0.99). rPfFPPS is expressed as its fractional activity; and risedronate concentrations were plotted on log scale. Data were fitted to Equation (2). Click here for file

Additional file 6 **Sequence logo analysis of the chain-length determination region.** All sequences containing the canonical DDxxD FARM motif were submitted to sequence logo analysis, as described in the Methods section. Total height of each position reflects overall sequence conservation at that column; height of each residue in a column reflects its proportion in relation to other possible residues for that column. Colors are for clarity, with aspartate in red, aromatic amino acids in blue, serine and cysteine in cyan, and all other amino acids in black. Click here for file

Additional file 7 **Proposed kinetic mechanism for rPfFPPS.** GGPP synthesis is proposed to follow a bi-bi ordered mechanism in an intricate system of parallel and consecutive reactions. Click here for file
