# Supplementary material for: Cloning and characterization of bifunctional enzyme farnesyl diphosphate/geranylgeranyl diphosphate synthase from Plasmodium falciparum
Source: Malar J. 2013 Jun 4;12:184. doi: 10.1186/1475-2875-12-184 (PMC3679732; doi:10.1186/1475-2875-12-184)
Supplement: Additional file 1 — Schematic representation of the integration of rPfFPPs-HA in genomic locus. A) Diagram illustrating the integration event by crossing-over and primers designed to detect this event (1, 2 and 3). Numbers 1 and 3 indicate the region where the primers have been designed for detecting the integration of the gene in locus. B) PCR detecting the integration of pFPP-HA in the genomic locus of P. falciparum using primers 1 and 3. C) Detecting the control PCR amplification of endogenous FPPS gene in both strains (transfected and 3D7) using the primers 1 and 2. (−) –negative control; (pFPPs-HA) – transfected strain; (3D7) – wild type strain. [file 1475-2875-12-184-S1.pdf]

File 1. Schematic representation of the integration of rPfFPPs-HA in genomic locus

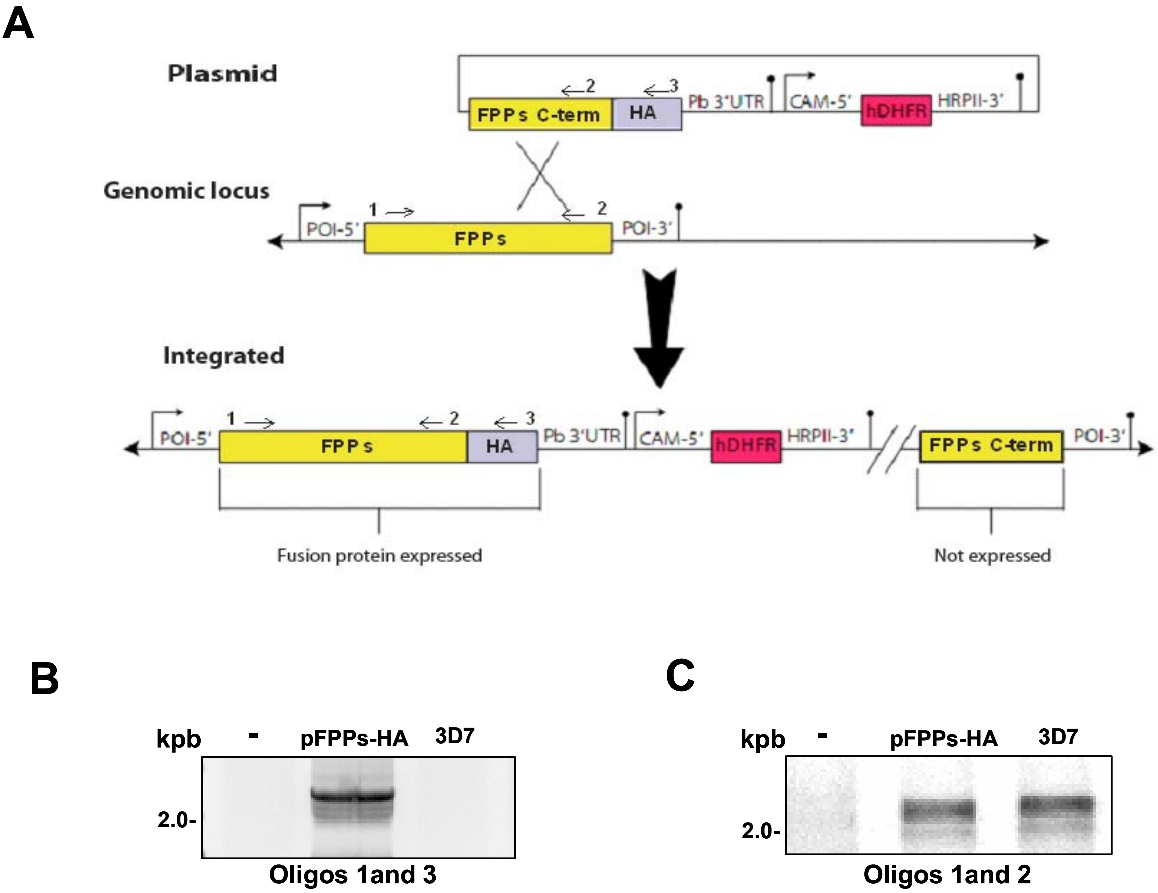

A) Diagram illustrating the integration event by crossing-over and primers designed to detect this event (1, 2 and 3). Numbers 1 and 3 indicate the region where the primers have been designed for detecting the integration of the gene in locus. **B**) PCR detecting the integration of pFPP-HA in the genomic locus of *P. falciparum* using primers 1 and 3. **C**) Detecting the control PCR amplification of endogenous FPPS gene in both strains (transfected and 3D7) using the primers 1 and 2. (-) – negative control; (**pFPPs-HA**) – transfected strain; (**3D7**) – wild type strain.
