# Supplementary material for: Cloning and characterization of bifunctional enzyme farnesyl diphosphate/geranylgeranyl diphosphate synthase from Plasmodium falciparum
Source: Malar J. 2013 Jun 4;12:184. doi: 10.1186/1475-2875-12-184 (PMC3679732; doi:10.1186/1475-2875-12-184)
Supplement: Additional file 2 — Table of organisms, accession numbers, and CLD region sequences analyzed. *Sequences characterized as bifunctional FPP/GPPS are highlighted in gray and use bold font, *Excluded from CLD analysis (X in red) were the sequences that either did not present the canonical DDxxD FARM motif or had rare insertions (see main text). [file 1475-2875-12-184-S2.pdf]

File 2. Table of organisms, accession numbers, and CLD region sequences analyzed

Accession numbers for sequences used in phylogenetic and CLD region analyses

Organism	Accession number	Taxonomic affiliation	CLD analysis	CLD region sequence
Acromyrmex echinatior	EGI61112.1	Metazoa		LMQAYFSIMDDIEDQSLFRRGQL
Acromyrmex echinatior	EGI61113.1	Metazoa		LMQAFLLVIDDIQDRSLIRRGQP
Acyrthosiphon pisum	XP_001950423.2	Metazoa		ILQAYQLVLDDIMDNAITRRGRP
Acyrthosiphon pisum	AAY33490.3	Metazoa		ILQAYQLVLDDIMDNAITRRGRP
Acyrthosiphon pisum	NP_001119633.3	Metazoa		ILQAYQLVLDDIMDNAITRRGRP
Aedes aegypti	XP_001663796.1	Metazoa		MFQAVFLICDDAMDGSQTRRGQP
Agaricus bisporus var. burnettii JB137-S8	EKM79467.1	Fungi		LLQSYFLVSDDIMDTSITRRGQP
Agrotis ipsilon	CAA08918.2	Metazoa	X	MFHTHQLLLNDIMEGAEMRRGAP
Ailuropoda melanoleuca	EFB14304.1	Metazoa		LLQAFFLVSDDIMDSSLTRRGQL
Ajellomyces capsulatus NAm1	XP_001543367.1	Fungi		FLQAFFLVSDDIMDSSVTRRGEP
Ajellomyces dermatitidis ER-3	EEQ89402.1	Fungi		FLQAFFLVSDDMMDSSITRRGEP
Albugo laibachii Nc14	CCA26954.1	Stramenopiles, Oomycetes		WLQASCLVIDDIMDQSAVRRRRP
Albugo laibachii Nc14	CCA25012.1	Stramenopiles, Oomycetes		LVQAFFLIADDIMDNSLMRRGQI
Algoriphagus sp. PR1	ZP_07722205.1	Bacteria, Bacteroidetes		VFHNFTLMHDDIMDQAPLRRGNA
Alisma orientale	ADV03674.1	Viridiplantae		WLQAYFLVLDDIMDNSHTRRGQP
Alisma plantago-aquatica	ADR83704.1	Viridiplantae		WLQAYFLVLDDIMDNSHTRRGQP
Allium sativum	AEG47693.1	Viridiplantae		WLQAYFLVLDDIMDNSHTRRGQP
Amphimedon queenslandica	XP_003386178.1	Metazoa		WLQAFFLVADDIMDQSLTRRGQP
Anolis carolinensis	XP_003227779.1	Metazoa		LLQAFFLVADDIMDGSLTRRGHP
Anopheles gambiae str. PEST	XP_308653.4	Metazoa		MLHSMFLIMDDVMDGSVTRRGQP
Anthonomus grandis	AAX78434.1	Metazoa		MIHSCFLVLDDIMDNSETRRGSL
Aphis fabae	AAY33488.2	Metazoa		ILQAYQLVLDDIMDNAITRRGRP
Aphis gossypii	ACT79808.1	Metazoa		ILQAYQLVLDDIMDNAITRRGRP
Aphis gossypii	ACT79809.3	Metazoa		ILQAYQLVLDDIMDNAITRRGRP
Apis florea	XP_003695132.1	Metazoa		ILQAVYIVMDDIVDHTDMRRNQP
Apis florea	XP_003695133.1	Metazoa		IMQAFHTMIDDIIDRASMRRNQS
Apis mellifera	XP_623586.2	Metazoa		ILQAVYIVMDDIIDHTDMRRNQP
Apis mellifera	XP_396224.4	Metazoa		IMQAFHTMIDDILDNANMRRNQP
Aquilaria microcarpa	ADH95185.1	Viridiplantae		WLQAYFLVLDDIMDNSHTRRGQP
Aquimarina agarilytica ZC1	ZP_10839501.1	Bacteria, Bacteroidetes		VFHNFSLVHDDIMDAAPLRRGKA
Arabidopsis lyrata subsp. lyrata	XP_002868080.1	Viridiplantae		WLQAYFLVLDDIMDNSVTRRGQP
Arabidopsis lyrata subsp. lyrata	XP_002863368.1	Viridiplantae		WLQAYFLVLDDIMDNSVTRRGQP
Arabidopsis thaliana	NP_193452.1	Viridiplantae		WLQAYFLVLDDIMDNSVTRRGQP
Arabidopsis thaliana	CAA53433.1	Viridiplantae		WLQAYFLVLDDIMDNSVTRRGQP
Aralia elata	ADK12004.1	Viridiplantae		WLQAYFLVLDDIMDSSHTRRGQP
Artemisia annua	AAD17204.1	Viridiplantae		WLQAYFLVLDDIMDESHTRRGQP
Arthrobotrys oligospora ATCC 24927	EGX52639.1	Fungi		LLQAFFLVSDDIMDGSKTRRGSP
Arthroderma benhamiae CBS 112371	XP_003015043.1	Fungi		LLQAFFLVSDDIMDSSITRRGEP
Arthroderma gypseum CBS 118893	XP_003174103.1	Fungi		LLQAFFLVSDDIMDSSITRRGEP
Arthroderma otae CBS 113480	XP_002848856.1	Fungi		LLQAFFLVSDDIMDSSITRRGEP
Ascaris suum	ADY46342.1	Metazoa		ILQAFFLIFDDVMDRSTTRRGKP
Ashbya gossypii ATCC 10895	NP_984739.1	Fungi		LLQAYFLVADDMMDKSITRRGQP
Aspergillus clavatus NRRL 1	XP_001276285.1	Fungi		LLQAFFLVSDDIMDSSITRRGQP

Aspergillus fumigatus A1163	EDP51096.1	Fungi		LLQAFFLVSDDMMDSSITRRGQP
Aspergillus kawachii IFO 4308	GAA84792.1	Fungi		TLQAYLLMHDDIMDNSSTRRGKP
Aspergillus kawachii IFO 4308	GAA84528.1	Fungi		LLQAFFLVSDDIMDGSITRRGQP
Aspergillus nidulans FGSC A4	XP_681281.1	Fungi		LLQAFFLVSDDLMDGSITRRGQP
Aspergillus niger CBS 513.88	XP_001397480.1	Fungi		LLQAYLLTHDDIMDNSSTRRGKP
Aspergillus niger CBS 513.88	XP_001400163.1	Fungi		LLQAFFLVSDDIMDGSITRRGQP
Aspergillus oryzae RIB40	XP_001822552.1	Fungi		LLQAFFLVSDDIMDSSITRRGQP
Aspergillus terreus NIH2624	XP_001215581.1	Fungi		MFQASYLVSDDIMDNSEYRRGKL
Aspergillus terreus NIH2624	XP_001217832.1	Fungi		LLQAFFLVSDDIMDSSITRRGQP
Atopobium rimae ATCC 49626	ZP_03568142.1	Bacteria, Actinobacteria		DFQSAALIHDDIADKSELRRGEP
Aureococcus anophagefferens	EGB06403.1	Stramenopiles, Pelagophyceae		MLQAWLLVADDMMDSSLTRRGQP
Auricularia delicata TFB-10046 SS5	EJD54366.1	Fungi		LLQATFLVTDDMMDSCELRRGQP
Auricularia delicata TFB-10046 SS5	EJD48967.1	Fungi		LLQAMFLVADDMMDSSVTRRGQD
Babesia bovis T2Bo	XP_001608674.1	Apicomplexa		LLQTAFLVADDIMDKSVMRRSNL
Babesia microti strain RI	CCF72499.1	Apicomplexa		LLQSAFIVADDIMDNGITRRGKA
Bacopa monnieri	ADV03080.1	Viridiplantae		WLQAYFLVLDDIMDNSHTRRGQP
Batrachochytrium dendrobatidis JAM81	EGF78709.1	Fungi		FLQAFFLISDDIMDGSLTRRGQP
Beauveria bassiana ARSEF 2860	EJP68529.1	Fungi		LLQAFFLVSDDIMDGSITRRGKP
Bizionia argentinensis JUB59	ZP_08819491.1	Bacteria, Bacteroidetes		VFHNFSLVHDDIMDDAPLRRGQE
Blakeslea trispora	AFC92797.1	Fungi		FLQAFFLVSDDIMDASITRRGQP
Blastocystis hominis	CBK22726.2	Stramenopiles		MLQGAFLIADDIMDEAEMRRGKP
Bombus impatiens	XP_003487810.1	Metazoa		LLQAFLLMVDDIQDHSEMRRNQP
Bombus terrestris	XP_003399196.1	Metazoa		LLQAFFLTIDDIQDHSEIRRNQP
Bombyx mori	NP_001093302.1	Metazoa		MLQAYLIMNDDIMDGSSTRRGVP
Bombyx mori	NP_001093301.1	Metazoa		MFQAYCIVLDDIMDGSSVRRGMP
Bombyx mori	NP_001036889.1	Metazoa	X	MFHTHQLLLNDIMEGTTMRRGVP
Bos taurus	NP_803463.1	Metazoa		LLQAFFLVSDDIMDSSLTRRGQT
Botryotinia fuckeliana B05.10	XP_001558276.1	Fungi		LLQAFFLVSDDIMDSSITRRGQP
Brachypodium distachyon	XP_003580726.1	Viridiplantae		WLQASALVLDDITDNAYTRRDNL
Brachypodium distachyon	XP_003569616.1	Viridiplantae		WLQAYFLVLDDIMDNSRTRRGKP
Brachypodium distachyon	XP_003569635.1	Viridiplantae		WLQAYFLVLDDIMDNSQTRRGQP
Brachypodium distachyon	XP_003567976.1	Viridiplantae		WLQAFFLVLDDIMDDSHTRRGQP
Branchiostoma floridae	XP_002602028.1	Metazoa		WLQAFFLVLDDIMDQSQTRRGQP
Brugia malayi	XP_001902397.1	Metazoa		LLQSFFLIEDDVMDEGVNRRGKP
Caenorhabditis brenneri	EGT38455.1	Metazoa		IIQSFYLIADDIMDNSETRRGKP
Caenorhabditis briggsae	XP_002640526.1	Metazoa		IIQSFYLIADDIMDNSETRRGKP
Caenorhabditis elegans	NP_493027.1	Metazoa		IIQSFYLIADDIMDNSETRRGKP
Caenorhabditis remanei	XP_003114889.1	Metazoa		IIQSFYLIADDIMDNSETRRGKQ
Callithrix jacchus	XP_002749779.1	Metazoa		LLQAFFLVTDDIMDSSLTRRGQI
Callithrix jacchus	XP_002760035.2	Metazoa		LLQAFFLVTDDIMDSSLTRRGQI
Camponotus floridanus	EFN60361.1	Metazoa		LAQAFFLLLDDIQDRSLFRRNQP
Candida albicans WO-1	EEQ45876.1	Fungi		LLQAYFLVADDMMDQSKTRRGQP
Candida dubliniensis CD36	XP_002418398.1	Fungi		LLQAYFLVADDMMDQSKTRRGQP
Candida glabrata CBS 138	XP_448787.1	Fungi		LLQAYFLVADDMMDKSITRRGQP

Candida orthopsilosis	CCG22223.1	Fungi		LLQAYFLVADDMMDQSKTRRGQP
Candida parapsilosis	CCE40357.1	Fungi		LLQAYFLVADDMMDQSETRRGQL
Candida tenuis ATCC 10573	EGV62778.1	Fungi		LLQGFFLVSDDIMDQSKTRRGQP
Candida tropicalis MYA-3404	XP_002547300.1	Fungi		LLQAYFLVADDMMDQSKTRRGQK
Canis lupus familiaris	XP_537252.2	Metazoa		LLQAFFLVSDDIMDSSLTRRGQI
Capnocytophaga canimorsus Cc5	YP_004740428.1	Bacteria, Bacteroidetes		VFHNFSLVHDDIMDKASLRRGQQ
Capnocytophaga gingivalis ATCC 33624	ZP_04058864.1	Bacteria, Bacteroidetes		IFHNFSLVHDDIMDAAPLRRGHP
Capnocytophaga ochracea DSM 7271	YP_003141950.1	Bacteria, Bacteroidetes		VFHNFSLIHDDIMDNASLRRGKP
Capnocytophaga sp. oral taxon 329 str. F0087	ZP_08450413.1	Bacteria, Bacteroidetes		VFHNFSLVHDDIMDNASLRRGKA
Capsaspora owczarzaki ATCC 30864	EFW40736.1	Opisthokonta incertae sedis		WLQAFFLVADDMMDQSQTRRGQP
Capsicum annuum	CAA59170.1	Viridiplantae		WLQAYFLVLDDIMDNSHTRRGQP
Catharanthus roseus	ADO95193.1	Viridiplantae		WLQAYFLVLDDIMDGSHTRRGQP
Cellulophaga lytica DSM 7489	YP_004262377.1	Bacteria, Bacteroidetes		VFHNFSLVHDDIMDDAPLRRGKA
Centella asiatica	AAV58896.1	Viridiplantae		WLQAYFLVLDDIMDGSHTRRGQP
Chaetomium globosum CBS 148.51	XP_001225389.1	Fungi		LLQAFFLVSDDIMDSSITRRGKP
Chaetomium thermophilum var. thermophilum DSM 1495	EGS21018.1	Fungi		LLQAFFLVSDDIMDGSITRRGKP
Chimonanthus praecox	ACJ38671.1	Viridiplantae		WLQAYFLVLDDIMDGSHTRRGQP
Chitinophaga pinensis DSM 2588	YP_003123335.1	Bacteria, Bacteroidetes		LFHNFTLVHDDIMDKAPLRRNQP
Chlamydomonas reinhardtii	XP_001693168.1	Viridiplantae		WLQAFFLVADDIMDGSITRRGQP
Chlorella variabilis	EFN59689.1	Viridiplantae		FLQAYFLVADDIMDSSITRRGQP
Choristoneura fumiferana	AAY26574.1	Metazoa		ILQGFLVMLDDIMDGSTTRRGVP
Choristoneura fumiferana	AAY26575.1	Metazoa	X	MFHTHQLLLNDIMEGTEMRRGAP
Ciona intestinalis	XP_002128313.1	Metazoa		ILQASFLVADDLMDQSKTRRGQA
Clavispora lusitaniae ATCC 42720	XP_002617167.1	Fungi		LLQAYFLVADDMMDQSKTRRGQP
Clonorchis sinensis	GAA57136.1	Metazoa		-----FLIHDDIIDNAPTRRNRT
Clonorchis sinensis	GAA49070.1	Metazoa		-----FLIHDDIIDNSPTRRNRT
Coccidioides immitis RS	XP_001242557.1	Fungi		LLQAFFLVSDDMMDTSITRRGEP
Coccomyxa subellipsoidea C-169	EIE24506.1	Viridiplantae		WLQAFFLVADDIMDNSITRRGQP
Cochliobolus eleusines	AFG34071.1	Fungi		LLQAFFLVSDDIMDSSKTRRGSP
Colletotrichum higginsianum	CCF34730.1	Fungi		LLQAFFLVSDDIMDSSITRRGKP
Coniophora puteana RWD-64-598 SS2	EIW77632.1	Fungi		LLQAFFLVSDDMMDDSTTRRGQP
Coprinopsis cinerea okayama7#130	XP_001840117.1	Fungi		LLQGFFLVSDDIMDASITRRGQP
Cordyceps militaris CM01	EGX94931.1	Fungi		LLQAFFLVSDDIMDSSITRRGKP
Crassostrea gigas	EKC20989.1	Metazoa		WFQAFFLVADDVMDSSITRRGKP
Cricetulus griseus	XP_003501631.1	Metazoa		LLQAFFLVSDDIMDSSLTRRGQI
Croceibacter atlanticus HTCC2559	YP_003715162.1	Bacteria, Bacteroidetes		VFHNFSLVHDDIMDDAPLRRGKD
Cryptococcus gattii WM276	XP_003194529.1	Fungi		LLQAYFLVADDIMDQSVTRRGQP
Cryptococcus neoformans var. neoformans JEC21	XP_571137.1	Fungi		LLQAYFLVADDIMDQSVTRRGQP
Cryptosporidium hominis TU502	XP_667626.1	Apicomplexa		AIQALILIADDIMDSGKFRRGAP
Cryptosporidium muris RN66	XP_002140353.1	Apicomplexa		ILQAFFLVGDDIMDGGEMRRGKV
Cryptosporidium parvum Iowa II	XP_625843.1	Apicomplexa		AIQALILIADDIMDSGKFRRGAP
Culex quinquefasciatus	XP_001841748.1	Metazoa	X	WFLCYAHVIFDDIVDNSKTRYGKP
Culex quinquefasciatus	XP_001841747.1	Metazoa		MFQSVFLICDDVMDGSQTRRGQP

Cyclocarya paliurus	ACY80695.1	Viridiplantae	WLQAYFLVLDDIMDNSVTRRGQP
Cymbidium goeringii	AFP19446.1	Viridiplantae	WLQAYFLVLDDIMDNSHTRRGQP
Dacryopinax sp. DJM-731 SS1	EJU05113.1	Fungi	LLQAFFLVSDDIMDTSITRRGQP
Danio rerio	AAH83515.1	Metazoa	LLQAFFLVADDIMDSSVTRRGQP
Daphnia pulex	EFX86316.1	Metazoa	MLQAFFLVADDIMDDSLTRRMKP
Debaryomyces hansenii CBS767	XP_460720.1	Fungi	LLQAYFLVADDMMDHSKTRRGQP
Dekkera bruxellensis AWRI1499	EIF46704.1	Fungi	LLQAYFLVADDIMDKSLTRRGHT
Dendroctonus jeffreyi	AAX78435.1	Metazoa	MVHAYVLILDDIMDGSETRRGAL
Dendroctonus ponderosae	AFI45068.1	Metazoa	MVHAYFLILDDIMDGSETRRGAL
Desmodus rotundus	JAA47240.1	Metazoa	LLQAFFLVTDDIMDSSLTRRGQI
Desulfurococcus mucosus DSM 2162	YP_004175949.1	Archaea, Crenarchaeota	LLQSYLLVHDDVMDRDEIRRGGP
Dichomitus squalens LYAD-421 SS1	EJF55972.1	Fungi	LLQAFFLVSDDMMDQSVTRRSQP
Dictyostelium discoideum AX4	XP_646923.1	Amoebozoa	FL-SFFLITDDIMDNGLTRRGEL
Dictyostelium discoideum AX4	XP_641989.1	Amoebozoa	IFQACYLVSDDIMDQSLKRRGKP
Dictyostelium discoideum AX4	XP_641990.1	Amoebozoa	WLQAFFLVADDIMDQSITRRGQP
Dictyostelium purpureum	XP_003286108.1	Amoebozoa	FL-SFFLVTDDIMDNGLTRRGEP
Dictyostelium purpureum	XP_003291032.1	Amoebozoa	FLQASLLIADDIMDGGIMRRDRP
Dictyostelium purpureum	XP_003283879.1	Amoebozoa	WLQSFFLVADDLMDSSITRRGQP
Drosophila ananassae	XP_001958742.1	Metazoa	MLQSFFIISDDVMDNSTTRRGQL
Drosophila erecta	XP_001976091.1	Metazoa	MLQSFFIISDDVMDNSITRRGQP
Drosophila grimshawi	XP_001987531.1	Metazoa	MLQSFFIISDDVMDNSTTRRGQK
Drosophila melanogaster	NP_477380.1	Metazoa	MLQSFFIISDDVMDNSTTRRGQP
Drosophila mojavensis	XP_002004330.1	Metazoa	MLQSFFIISDDVMDNSTTRRGQK
Drosophila pseudoobscura pseudoobscura	XP_001361556.1	Metazoa	MLQSFFIISDDVMDNSTTRRGQP
Drosophila sechellia	XP_002033374.1	Metazoa	MLQSFFIISDDVMDNSTTRRGQP
Drosophila simulans	XP_002081024.1	Metazoa	MLQSFFIISDDVMDNSTTRRGQP
Drosophila virilis	XP_002059798.1	Metazoa	MLQSFFIISDDVMDNSTTRRGQK
Drosophila willistoni	XP_002066117.1	Metazoa	MLQSFFIMSDDVMDNSTTRRGQL
Drosophila yakuba	XP_002090289.1	Metazoa	MLQSFFIISDDVMDNSTTRRGQP
Edhazardia aedis USNM 41457	EJW01916.1	Fungi	ILQAYFLVTDDIIDNSETRRGKR
Eimeria tenella	ETH_00019475	Apicomplexa	LLQAAFLVADDQMDGAFTRRGKA
Eleutherococcus senticosus	AEY77151.1	Viridiplantae	WLQAYFLVLDDIMDSSHTRRGQP
Emticicia oligotrophica DSM 17448	YP_006872642.1	Bacteria, Bacteroidetes	VFHNFTLMHDDIMDAAPLRRGQP
Encephalitozoon hellem ATCC 50504	XP_003888424.1	Fungi	LLQAALLIVDDLMDNSEIRRGRP
Equus caballus	XP_003365053.1	Metazoa	LLQAFFLVSDDIMDSSLTRRGQV
Eremothecium cymbalariae DBVPG#7215	XP_003648213.1	Fungi	LLQAYFLVADDMMDKSITRRGQP
Eucommia ulmoides	BAB16687.2	Viridiplantae	WLQACALVLDDIMDSSHTRRGQM
Eucommia ulmoides	BAB60822.1	Viridiplantae	WLQAYFLVLDDIMDSSHTRRGQP
Euphorbia pekinensis	ACN63187.1	Viridiplantae	WLQAYFLVLDDIMDGSHTRRGQP
Exophiala dermatitidis NIH/UT8656	EHY54301.1	Fungi	LLQAFFLVSDDIMDASITRRGEP
Felis catus	XP_003999685.1	Metazoa	LLQAFFLVSDDIMDSSLTRRGQI
Fibroporia radiculosa	CCM04365.1	Fungi	LLQAFFLVSDDMMDSSITRRDQP
Filobasidiella depauperata	ACZ80672.1	Fungi	LLQAYFLVADDIMDQSVTRRGQP
Flavobacterium columnare ATCC 49512	YP_004942525.1	Bacteria, Bacteroidetes	VFHNFSLVHDDIMDNAPLRRGNE
Flavobacterium indicum GPTSA100-9	YP_005356285.1	Bacteria, Bacteroidetes	VFHNFSLVHDDIMDDAPLRRGKK

Flavobacterium johnsoniae UW101	YP_001193614.1	Bacteria, Bacteroidetes	VFHNFSLVHDDIMDDAPLRRGQV
Flexibacter litoralis DSM 6794	YP_006434379.1	Bacteria, Bacteroidetes	VFHNFTLLHDDIMDNAPIRRGQP
Fomitiporia mediterranea MF3/22	EJC99079.1	Fungi	LLQAFFLVSDDMMDQSITRRGQP
Fusarium oxysporum Fo5176	EGU79834.1	Fungi	LLQAFFLVSDDIMDSSITRRGQP
Fusarium pseudograminearum CS3096	EKJ68072.1	Fungi	LLQAFFLVSDDIMDSSITRRGQP
Gaeumannomyces graminis var. tritici R3-111a-1	EJT77310.1	Fungi	LLQAFFLVSDDIMDSSITRRGQP
Galbibacter sp. ck-I2-15	ZP_11167363.1	Bacteria, Bacteroidetes	IFHNFSLVHDDIMDDAPLRRGKQ
Ganoderma lucidum	ACB37020.1	Fungi	FLQAFFLVSDDMMDQSVTRRGQP
Gentiana lutea	BAA88844.1	Viridiplantae	WLQAYFLVLDDIMDGSHTRRSQP
Giardia intestinalis ATCC 50581	EES99536.1	Diplomonadida	TLQAAFLMLDDVIDHSTVRRGKP
Giardia lamblia ATCC 50803	XP_001709477.1	Diplomonadida	ILQGAFLMIDDVIDHSTIRRGKP
Giardia lamblia P15	EFO63579.1	Diplomonadida	ILQGAFLMIDDVIDHSTIRRGKP
Gibberella zeae PH-1	XP_386960.1	Fungi	LLQAFFLVSDDIMDSSITRRGQP
Gilbertella persicaria	CAR64517.1	Fungi	FLQAFFLVSDDIMDASITRRGQP
Gillisia sp. CBA3202	ZP_11223116.1	Bacteria, Bacteroidetes	VFHNFSLVHDDIMDDAPLRRGKA
Ginkgo biloba	AAR27053.1	Viridiplantae	WLQAYFLVLDDIMDGSHTRRGQP
Glarea lozoyensis 74030	EHL02094.1	Fungi	LLQAFFLVSDDIMDSSITRRGAP
Glomerella graminicola M1.001	EFQ27995.1	Fungi	LLQAFFLVSDDIMDTSITRRGKP
Glossina morsitans morsitans	ADD19099.1	Metazoa	LLQCFLVLNDDIMDNSTTRRGQT
Glycine max	NP_001242101.1	Viridiplantae	WLQAYFLVLDDIMDNSHTRRGQP
Glycyrrhiza uralensis	ADE18770.1	Viridiplantae	WLQAYFLVLDDIMDNSHTRRGQP
Gossypium arboreum	CAA72793.1	Viridiplantae	WLQAYFLVLDDIMDSSHTRRGQP
Gramella forsetii KT0803	YP_863095.1	Bacteria, Bacteroidetes	IFHNFSLVHDDIMDDAPLRRGKE
Grosmannia clavigera kw1407	EFW99779.1	Fungi	LLQFAILASDDIVDGDLWRRGRW
Grosmannia clavigera kw1407	EFW99652.1	Fungi	LLQAFFLISDDIMDSSITRRGNP
Haliscomenobacter hydrossis DSM 1100	YP_004445412.1	Bacteria, Bacteroidetes	IFHNFSLVHDDVMDAAPLRRGQA
Harpegnathos saltator	EFN82314.1	Metazoa	LLQGFLLILDDIEDRSLIRRKQL
Harpegnathos saltator	EFN90138.1	Metazoa	LLHAFFIMIDDIQDRSQFRRNQP
Hedychium coccineum	AER12202.1	Viridiplantae	WLQAYFLVLDDIMDDSVTRRGQP
Heterocephalus glaber	EHA99577.1	Metazoa	LLQAFFLVADDIMDSSLTRRGRA
Hevea brasiliensis	AAM98379.1	Viridiplantae	WLQAYFLVLDDIMDSSHTRRGQP
Homo sapiens	CAA87327.1	Metazoa	LLQAFFLVADDIMDSSLTRRGQI
Homo sapiens	AAA52423.1	Metazoa	LLQAFFLVADDIMDSSLTRRGQT
Hordeum vulgare subsp. vulgare	BAJ84838.1	Viridiplantae	WLQASALVLDDITDNAYTRRDNL
Hordeum vulgare subsp. vulgare	BAJ84778.1	Viridiplantae	WLQAYFLVLDDIMDNSQTRRGQP
Hordeum vulgare subsp. vulgare	BAJ90047.1	Viridiplantae	WLQAFFLVLDDIMDDSHTRRGQP
Hordeum vulgare subsp. vulgare	BAJ95824.1	Viridiplantae	FLQAFFLIADDIMDASVTRRGQP
Humulus lupulus	BAB40665.1	Viridiplantae	WLQAYFLVLDDIMDNSVTRRGQP
Huperzia carinata	AFO53558.1	Viridiplantae	WLQAYYLVLDDIMDNSVTRRGQA
Huperzia serrata	AFO53556.1	Viridiplantae	WLQAYYLVLDDIMDNSVTRRGQP
Imtechella halotolerans K1	ZP_09998137.1	Bacteria, Bacteroidetes	VFHNFSLVHDDIMDGAPLRRGKP
Ips confusus	ACV13206.1	Metazoa	IIQAYFCMLDDIMDDSDTRRGKP
Ips pini	AAX55632.1	Metazoa	IIQAYFCMLDDIMDDSDTRRGKP

Ips pini	AAX55631.1	Metazoa		MLHTYFLIIDDIIDHSDTRRGAI
Ixodes scapularis	XP_002408650.1	Metazoa		MLQSYFLILDDIMDGSPVRRGRP
Joostella marina DSM 19592	ZP_10106368.1	Bacteria, Bacteroidetes		MFHNFSLVHDDIMDDAPLRRGKE
Kazachstania africana CBS 2517	XP_003958211.1	Fungi		LLQAYFLVADDMMDQSITRRGQP
Kazachstania naganishii CBS 8797	CCK69214.1	Fungi		LLQAYFLVADDMMDQSITRRGQP
Kluyveromyces lactis NRRL Y-1140	XP_451300.1	Fungi		LLQAYFLVADDMMDQSITRRGQP
Komagataella pastoris GS115	XP_002490436.1	Fungi		LLQAYFLVADDMMDQSITRRGQP
Laccaria bicolor S238N-H82	XP_001879398.1	Fungi		LLQAFFLVSDDIMDSSITRRSQP
Lachancea thermotolerans	XP_002554410.1	Fungi		LLQAYFLVADDMMDKSITRRGQP
Lactarius chrysorrheus	BAD15361.1	Fungi		WMQAYFLVSDDIMDASITRRGQP
Leishmania braziliensis MHOM/BR/75/M2904	XP_001565042.1	Kinetoplastida		MLQAHFLVEDDIMDNSKTRRGRP
Leishmania donovani	ABI16061.1	Kinetoplastida		MLQAHFLVEDDIMDHSKTRRGKP
Leishmania infantum JPCM5	XP_003392505.1	Kinetoplastida		MLQAHFLVEDDIMDHSKTRRGKP
Leishmania major strain Friedlin	XP_001683287.1	Kinetoplastida		MLQAHFLVEDDIMDHSKTRRGKP
Leishmania mexicana MHOM/GT/2001/U1103	XP_003875590.1	Kinetoplastida		MLQAHFLVEDDIMDHSKTRRGKP
Leptosphaeria maculans JN3	XP_003843669.1	Fungi		LLQAFFLVSDDIMDSSKTRRGSP
Lilium longiflorum	ADZ57167.1	Viridiplantae		WLQAYFLVLDDIMDNSHTRRGQL
Loa loa	XP_003141856.1	Metazoa		LLQSFFLIEDDVMDEGVNRRGKP
Lodderomyces elongisporus NRRL YB-4239	XP_001527130.1	Fungi		LLQAYFLVADDMMDQSKTRRGQP
Lotus japonicus	AFK38522.1	Viridiplantae		WLQAYFLVLDDIMDNSHTRRGQP
Loxodonta africana	XP_003414979.1	Metazoa		LLQSFFLVLDDIMDSSLTRRGQL
Macaca fascicularis	EHH60828.1	Metazoa		LLQAFFLVADDIMDSSLSPQGQI
Macaca fascicularis	EHH50345.1	Metazoa		LLQAFFLVTDDIMDSSLTRRGQI
Macaca mulatta	XP_001097788.1	Metazoa		LLQAFFLVADDIMDSSLSPQGQI
Macaca mulatta	AFE67702.1	Metazoa		LLQAFFLVTDDIMDSSLTRRGQI
Macaca mulatta	EHH15320.1	Metazoa		LLQAFFLVTDDIMDSSLTRRGQI
Macrophomina phaseolina MS6	EKG15848.1	Fungi		LLQASLLVNDDIMDGGKTRRGRT
Macrophomina phaseolina MS6	EKG13554.1	Fungi		LLQAYFLVVDDIMDGSQTRRGQP
Macrophomina phaseolina MS6	EKG18873.1	Fungi		LLQAFFLVSDDIMDSSKTRRGSP
Magnaporthe oryzae 70-15	XP_003711082.1	Fungi		LLQAFFLVSDDIMDSSITRRGQP
Magnolia chapensis	ACS74708.1	Viridiplantae		WLQAYFLVLDDIMDGSHTRRGQP
Malassezia globosa CBS 7966	XP_001732045.1	Fungi		LLQAYFLVADDMMDASITRRGHP
Malus x domestica	AAM08927.1	Viridiplantae		WLQAFFLVLDDIMDGSHTRRGQP
Marssonina brunnea f. sp. 'multigermtubi' MB_m1	EKD14727.1	Fungi		LLQAFFLVSDDIMDSSITRRGAP
Matricaria chamomilla var. recutita	ABS11699.1	Viridiplantae	X	WLQAYFLVLGDIMDESHTRRGQP
Medicago sativa	ADC32809.1	Viridiplantae		WLQAYFLVLDDIMDNSHTRRGQP
Medicago truncatula	XP_003594327.1	Viridiplantae		WLQAYFLVLDDIMDNSHTRRGQP
Megachile rotundata	XP_003705311.1	Metazoa		LLQAFLLVIDDIQDQSTIRRNAP
Megoura viciae	AAY33489.2	Metazoa		ILQAYQLVLDDIMDNAITRRGRP
Melampsora larici-populina 98AG31	EGG11672.1	Fungi		LLQAYFLVADDMMDQSVTRRGQP
Mentha x piperita	AAK63847.1	Viridiplantae		WLQAYFLVLDDIMDNSHTRRGQP
Metarhizium acridum CQMa 102	EFY87987.1	Fungi		LLQAFFLVSDDMMDGSITRRGKP
Metarhizium anisopliae ARSEF 23	EFY95899.1	Fungi		MLGAHYLILDDIMDDSTTRRGEP

Metarhizium anisopliae ARSEF 23	EFY95357.1	Fungi		LLQAFFLVSDDMMDGSITRRGKP
Metaseiulus occidentalis	XP_003748623.1	Metazoa		MFQAHYLILDDIMDKSITRRNRK
Methanothermobacter marburgensis str. Marburg	YP_003849447.1	Archaea, Crenarchaeota		LIHTFSLIHDDIMDDDEMRRGEP
***Methanothermobacter thermautotrophicus* str. Delta H**	**NP_275193.1**	**Archaea, Crenarchaeota**		**LIHTFSLIHDDIMDDDEIRRGEP**
Meyerozyma guilliermondii ATCC 6260	EDK37369.2	Fungi		MLQAFFLVADDIMDKAEKRRGHP
Micromonas pusilla CCMP1545	XP_003062388.1	Viridiplantae		WLQAAFLVWDDIMDESVTRRGQP
Microscilla marina ATCC 23134	ZP_01687869.1	Bacteria, Bacteroidetes		VFHNFTLVHDDIMDEAPLRRGKP
Millerozyma farinosa CBS 7064	CCE84448.1	Fungi		LLQAYFLVADDIMDKSKTRRGQK
Mixia osmundae IAM 14324	GAA95763.1	Fungi		LLQAYFLVADDMMDQSITRRGQP
Monodelphis domestica	XP_001373435.1	Metazoa		-VQAFFLVADDIMDSSLTRRGQP
Monosiga brevicollis MX1	XP_001743857.1	Choanoflagellida		WLQAFFLVADDIMDGSVTRRGQP
Mucor circinelloides f. lusitanicus	CAD42869.1	Fungi		FLQAFFLVSDDIMDASITRRGQP
Mus musculus	BAE38529.1	Metazoa		LLQAFFLVSDDIMDSSLTRRGQI
Musa acuminata	AAL82595.1	Viridiplantae		WLQAYFLVLDDIMDNSHTRRGQL
Mustela putorius furo	AER98526.1	Metazoa		LLQAFLLVCDDIMDSSLTRRGQI
Myceliophthora thermophila ATCC 42464	XP_003661689.1	Fungi		LLQAFFLVSDDIMDGSITRRGKP
Mythimna unipuncta	AAY33487.1	Metazoa	X	MFHTHQLLLNDIMEGTEMRRGAP
Myzus persicae	ABY19313.1	Metazoa		ILQAYQLVMDDLMDNAITRRGRP
Myzus persicae	ABY19312.1	Metazoa		ILQAYQLVLDDIMDNAITRRGRP
Myzus persicae	AAY33491.1	Metazoa		ILQAYQLVLDDIMDNAITRRGRP
Naegleria gruberi	XP_002682719.1	Heterolobosea		FLQAFFLIADDIMDASVTRRGQP
Nasonia vitripennis	XP_001604464.2	Metazoa		MVQAFFLVLDDVMDHSESRRGQP
Naumovozyma castellii CBS 4309	XP_003675288.1	Fungi		LLQAYFLVADDMMDKSITRRGQP
Naumovozyma dairenensis CBS 421	XP_003667492.1	Fungi		LLQAYFLVADDMMDKSITRRGQP
Nectria haematococca mpVI 77-13-4	XP_003050078.1	Fungi		LLQAFFLVSDDIMDSSITRRGQP
Nematostella vectensis	XP_001633044.1	Metazoa		WFQAFFLVADDIMDQSMTRRGQP
Neosartorya fischeri NRRL 181	XP_001266284.1	Fungi		LLQAFFLVSDDMMDSSITRRGQP
Neospora caninum Liverpool	NCLIV_048200	Apicomplexa		LLQSCFLVMDDVMDHSITRRGKP
Neurospora crassa OR74A	XP_961541.1	Fungi		LLQAFFLVSDDIMDSSITRRGKP
Nicotiana tabacum	ACU51163.1	Viridiplantae		WLQAYFLVLDDIMDNSHTRRGQP
Nomascus leucogenys	XP_003259483.1	Metazoa		LVRG-LLVADDIMDSSLTRRGQI
Nosema ceranae BRL01	XP_002995273.1	Fungi		LLQSAFIVADDVMDESLIRRGVE
Nosema ceranae BRL01	XP_002996129.1	Fungi		LFHASFILSDDIVDNSLIRRNKP
Ogataea parapolymorpha DL-1	EFW97329.1	Fungi		LLQAYFLVADDMMDKSITRRGQP
Oikopleura dioica	CBY39100.1	Metazoa		CLQTCFLVVDDIMDSSVTRRGRA
Olea europaea	AFS28706.1	Viridiplantae		WLQAYFLVLDDIMDNSHTRRGQP
Oreochromis niloticus	XP_003450942.1	Metazoa		LLQAFFLVADDIMDGSVTRRGQP
Oryza sativa Indica Group	CAH66696.1	Viridiplantae		WFQACALLLDDIMDDSHTRRDQI
Oryza sativa Indica Group	EEC71327.1	Viridiplantae		WLQAYFLVLDDIMDNSQTRRGKP
Oryza sativa Indica Group	EEC79629.1	Viridiplantae		WLQAYFLVLDDIMDDSHTRRGQP
Oryza sativa Indica Group	EAY75519.1	Viridiplantae		WLQAYFLVLDDIMDNSQTRRGQP
Oryza sativa Japonica Group	NP_001054121.1	Viridiplantae		WFQACALLLDDIMDDSHTRRDQI
Oryza sativa Japonica Group	NP_001054122.1	Viridiplantae		WLQASALVLDDITDNAYTRRDNL

Oryza sativa Japonica Group	EEE55234.1	Viridiplantae	WLQAYFLVLDDIMDNSQTRRGKP
Oryza sativa Japonica Group	NP_001056204.1	Viridiplantae	WLQAYFLVLDDIMDDSHTRRGQP
Oryza sativa Japonica Group	NP_001044000.1	Viridiplantae	WLQAYFLVLDDIMDNSQTRRGQP
Ostreococcus lucimarinus CCE9901	XP_001416643.1	Viridiplantae	FLQAYFLVADDIMDESVTRRGQP
Ostreococcus tauri	XP_003078152.1	Viridiplantae	FLQAYFLVADDIMDESVTRRGQP
Otolemur garnettii	XP_003795330.1	Metazoa	LLQAFFLVADDIMDSSLTRRGKI
Pan troglodytes	JAA09023.1	Metazoa	LLQAFFLVADDIMDSSLTRRGQI
Panax ginseng	AAY87903.1	Viridiplantae	WLQAYFLVLDDIMDSSHTRRGQP
Panax notoginseng	AAY53905.1	Viridiplantae	WLQAYFLVLDDIMDSSHTRRGQP
Panax quinquefolius	ADJ68004.1	Viridiplantae	WLQAYFLVLDDIMDSSHTRRGQP
Papilio polytes	BAM20475.1	Metazoa	LLMSYILILDDIEDGATSRNGLP
Papio anubis	XP_003892829.1	Metazoa	LLQAFFLVTDDIMDSSLTRRGQI
Papio anubis	XP_003892831.1	Metazoa	LLQAFFLVTDDIMDSSLTRRGQI
Paracoccidioides brasiliensis Pb03	EEH23256.1	Fungi	LLQAFFLVSDDMMDGSHTRRGEP
Pediculus humanus corporis	XP_002428407.1	Metazoa	ILQAKLLIADDIMDNSLTRRGRP
Penicillium aethiopicum	ADI24929.1	Fungi	LLQATMLVLDDIMDGSPTRRGKP
Penicillium brevicompactum	AEL21379.1	Fungi	LLQAFFLVSDDMMDSSITRRGQP
Penicillium chrysogenum Wisconsin 54-1255	XP_002568534.1	Fungi	LLQAFFLVSDDMMDSSITRRGQP
Penicillium marneffei ATCC 18224	XP_002147607.1	Fungi	LLQAFFLVSDDIMDGSITRRGQP
Perkinsus marinus ATCC 50983	XP_002776390.1	Alveolata	FLQASFLMFDDVMDQSETRRGST
Perkinsus marinus ATCC 50983	XP_002769627.1	Alveolata	ILQSHFLTLDDVMDSSSTRRGKP
Phaeodactylum tricornutum CCAP 1055/1	XP_002184024.1	Stramenopiles, Bacillariophyta	FLQAFFLVADDVMDDSQTRRGQP
Phaeosphaeria nodorum SN15	XP_001805566.1	Fungi	LLQAFFLVSDDIMDSSKTRRGSP
Phanerochaete carnosa HHB-10118-sp	EKM49650.1	Fungi	LLQAFFLVSDDVMDSSITRRDQP
Phaseolus lunatus	BAC53873.2	Viridiplantae	WLQAYFLVLDDIMDNSHTRRGQP
Physcomitrella patens subsp. patens	XP_001768279.1	Viridiplantae	WLQGYFLVMDDIMDNSVTRRGQP
Phytophthora infestans T30-4	XP_002901780.1	Stramenopiles, Oomycetes	WLQAFFLIADDIMDESITRRGQP
Phytophthora sojae	EGZ24043.1	Stramenopiles, Oomycetes	WLQAFFLVADDIMDESITRRGQP
Picea abies	ACA21460.1	Viridiplantae	WLQAYFLILDDIMDSSHTRRGQP
Picea sitchensis	ACN40171.1	Viridiplantae	WLQAYFLILDDIMDSSHTRRGQP
Plagiochasma appendiculatum	AFM78687.1	Viridiplantae	WLQGYFLVEDDIMDGSVTRRGQP
Plasmodium berghei strain ANKA	XP_677929.1	Apicomplexa	ILQASFLVADDIMDKGETRRNKH
Plasmodium chabaudi chabaudi	XP_740118.1	Apicomplexa	ILQASFLVADDIMDKGETRRNKH
Plasmodium cynomolgi strain B	GAB66562.1	Apicomplexa	ILQASFLVADDIMDKGETRRNKY
***Plasmodium falciparum* 3D7**	**XP_001347966.2**	**Apicomplexa**	**ILQASFLVADDIMDKGETRRNKH**
Plasmodium knowlesi strain H	XP_002259291.1	Apicomplexa	ILQASFLVADDIMDKGEKRRKKY
Plasmodium vivax Sal-1	XP_001615401.1	Apicomplexa	ILQAAFLVADDIMDKGETRRNKY
Podospora anserina S mat+	XP_001911873.1	Fungi	LLQAFFLVSDDIMDSSITRRGKP
Polaribacter irgensii 23-P	ZP_01117430.1	Bacteria, Bacteroidetes	VFHNFTLVHDDIMDAAPLRRGKP
Polaribacter sp. MED152	ZP_01053322.1	Bacteria, Bacteroidetes	VFHNFTLVHDDIMDDAPLRRGKA
Pongo abelii	NP_001125620.1	Metazoa	LLQAFFLVADDIMDSSLTRRGQI
Populus trichocarpa	ABK95166.1	Viridiplantae	WLQAYFLVLDDIMDSSHTRRGQP
Prevotella micans F0438	ZP_09590479.1	Bacteria, Bacteroidetes	TYHNYTLLHDDLMDKAPLRRGKP

Psychroflexus torquis ATCC 700755	YP_006867956.1	Bacteria, Bacteroidetes		IFHNFSLVHDDIMDDAPLRRGQQ
Puccinia graminis f. sp. tritici CRL 75-36-700-3	XP_003307564.1	Fungi		LLQAYFLVADDMMDHSLTRRGQP
Punctularia strigosozonata HHB-11173 SS5	EIN04079.1	Fungi		LLQAFFLVSDDLMDSSVTRRGQP
Pyrenophora teres f. teres 0-1	XP_003300141.1	Fungi		FLQAFFLVSDDIMDSSKTRRGNP
Pyrenophora tritici-repentis Pt-1C-BFP	XP_001941414.1	Fungi		FLQAFFLVSDDIMDSSKTRRGNP
Rattus norvegicus	NP_114028.1	Metazoa		LLQAFFLVLDDIMDSSHTRRGQI
Rhizopus delemar RA 99-880	EIE89971.1	Fungi		FLQAFFLVSDDIMDASITRRGQP
Rhopalosiphum padi	AEK32004.1	Metazoa		ILQAYQLVLDDIMDNAITRRGRA
Rhopalosiphum padi	AEK32003.1	Metazoa		ILQAYQLVLDDIMDNAITRRGRP
Ricinus communis	XP_002522802.1	Viridiplantae		WLQACAVVLDDIMDNSHTRRGRP
Ricinus communis	XP_002534338.1	Viridiplantae		WLQAYFLVLDDIMDGSHTRRGQP
Saccharomyces arboricola H-6	EJS43215.1	Fungi		LLQAYFLVADDMMDKSITRRGQP
Saccharomyces cerevisiae Kyokai no. 7	GAA24199.1	Fungi		LLQAYFLVADDMMDKSITRRGQP
Saccharomyces kudriavzevii IFO 1802	EJT43164.1	Fungi		LLQAYFLVADDMMDKSITRRGQP
Saimiri boliviensis boliviensis	XP_003927593.1	Metazoa		LLQAFFLVTDDIMDSFLTRQRQL
Saimiri boliviensis boliviensis	XP_003937858.1	Metazoa		LLQAFFLVTDDIMDSSLTRRGQL
Salmo salar	NP_001133850.1	Metazoa		LLQAFFLVADDIMDASVTRRGQP
Salvia miltiorrhiza	ABV08819.1	Viridiplantae		WLQAYFLVLDDIMDNSHTRRGQP
Santalum album	AEY80378.1	Viridiplantae		WLQAYFLVLDDIMDGSHTRRGQP
Sarcophilus harrisii	XP_003772978.1	Metazoa	X	LLQVFFYITDDIMNFSLTS-GES
Sarcophilus harrisii	XP_003768123.1	Metazoa		LLQAFFLVADDIMDSSLTRRGQP
Scheffersomyces stipitis CBS 6054	XP_001384571.1	Fungi		LLQAYFLVADDMMDQSKTRRGQP
Schistosoma japonicum	CAX73492.1	Metazoa		LLHAGFLVLDDIIDNSTLRRGQP
Schistosoma mansoni	CCD78373.1	Metazoa		LLHAGFLVLDDIIDNSTLRRGQP
Schizophyllum commune H4-8	XP_003032976.1	Fungi		LLQAFFLVSDDIMDSSITRRGQP
Schizosaccharomyces japonicus yFS275	XP_002171857.1	Fungi		MLQGSFLVADDIMDKSLTRRGRP
Schizosaccharomyces japonicus yFS275	XP_002174505.1	Fungi		LLQAFFLVADDMMDESKTRRGQP
Schizosaccharomyces pombe 972h-	NP_595334.1	Fungi		ILQGCFLMADDIMDQSIKRRGLD
Schizosaccharomyces pombe 972h-	NP_593299.1	Fungi		LLQSFFLIADDIMDASKTRRGQP
Sclerotinia sclerotiorum 1980	XP_001591034.1	Fungi		LLQAFFLVSDDIMDGSITRRGQP
Selaginella moellendorffii	XP_002991307.1	Viridiplantae		WLQAYFLVMDDIMDNSHTRRGKP
Serpula lacrymans var. lacrymans S7.9	EGO22908.1	Fungi		LLQAFFLVSDDMMDQSITRRSQP
Slackia heliotrinireducens DSM 20476	YP_003144326.1	Bacteria, Actinobacteria		HFHTAALIHDDIADEATLRRGEP
Solanum lycopersicum	NP_001234068.1	Viridiplantae		WLQAYFLVLDDIMDGSHTRRGQP
Solenopsis invicta	EFZ18629.1	Metazoa		LTQAAAIMIDDLQDQSLFRRGNP
Solitalea canadensis DSM 3403	YP_006255492.1	Bacteria, Bacteroidetes		VFHNFTLVHDDIMDNAPIRRGKA
Sordaria macrospora k-hell	XP_003349654.1	Fungi		LLQAFFLVSDDIMDSSITRRGKP
Sorghum bicolor	XP_002448677.1	Viridiplantae	X	FLRAYDRLRDELLDDDSCELTDEA
Sorghum bicolor	XP_002456217.1	Viridiplantae		WLQAYFLVLDDIMDNSQTRRGQP
Sorghum bicolor	XP_002441458.1	Viridiplantae		WLQAFFLVLDDIMDESHTRRGQP
Spathaspora passalidarum NRRL Y-27907	EGW33042.1	Fungi		LLQAYFLVADDMMDQSKTRRGQP
Sporisorium reilianum SRZ2	CBQ71652.1	Fungi		LLQAYFLVADDMMDASVTRRGQP
Staphylothermus hellenicus DSM 12710	YP_003668340.1	Archaea, Crenarchaeota		FLQSYLLVHDDIMDEDEIRRGGP

Staphylothermus marinus F1	YP_001040510.1	Archaea, Crenarchaeota		FLQSYLLVHDDIMDEDEIRRGGP
Stereum hirsutum FP-91666 SS1	EIM85784.1	Fungi		FLQAHFLVADDLMDQSLTRRGQP
Strongylocentrotus purpuratus	XP_781908.3	Metazoa		WLQAYFLIADDMMDQSKTRRGQP
Sus scrofa	NP_001172060.1	Metazoa		LLQAFFLVADDIEDSSLTRRGQT
Taeniopygia guttata	XP_002200183.1	Metazoa		LFQAFFLVADDIMDASLTRRGQL
Tagetes erecta	AEY78646.1	Viridiplantae		WLQAFILVLDDIMDGSHTRRGQP
Takifugu rubripes	XP_003966330.1	Metazoa		LLQAFFLVADDIMDASVTRRGQP
Talaromyces stipitatus ATCC 10500	XP_002481764.1	Fungi		LLQAFFLVSDDIMDGSITRRGQP
Tanacetum cinerariifolium	ADO17798.1	Viridiplantae		WLQAFLLIHDDIMDGSHTRRGQP
Taxus x media	AAS19931.1	Viridiplantae		WLQAYFLVLDDIMDGSHTRRGQP
Tetraodon nigroviridis	CAG11850.1	Metazoa		LLQAFFLVADDIMDASVTRRGQP
Tetrapisispora blattae CBS 6284	CCH60375.1	Fungi		LLQAYFLVADDMMDHSLTRRGQP
Tetrapisispora phaffii CBS 4417	XP_003685858.1	Fungi		LLQAYFLVADDMMDKSITRRGQP
Tetropium fuscum	AFR31785.1	Metazoa		LLRGFELIVDDIVDNAETRRNAP
Thalassiosira oceanica	EJK75002.1	Stramenopiles, Bacillariophyta		WLQAFFLVADDIMDASITRRGSP
Thalassiosira pseudonana CCMP1335	XP_002294044.1	Stramenopiles, Bacillariophyta		WLQAFFLVADDIMDGSITRRGNP
Theileria annulata strain Ankara	XP_955531.1	Apicomplexa		LLQTSFLVADDIIDKSTKRRSNT
Theileria orientalis strain Shintoku	BAM40763.1	Apicomplexa		LLQTSFLVADDIMDQSTKRRSNT
Theileria parva strain Muguga	XP_762981.1	Apicomplexa		LLQTSFLVADDIIDKSLKRRSNT
Thermosipho africanus TCF52B	YP_002335290.1	Bacteria, Thermotogae		IMHCFLLIHDDIMDRSALRRGLP
Thermosphaera aggregans DSM 11486	YP_003650291.1	Archaea, Crenarchaeota		LLQSYLLVHDDIMDMDELRRGGP
Thielavia terrestris NRRL 8126	XP_003649209.1	Fungi		LLQAFFLVSDDIMDGSITRRGKP
Torulaspora delbrueckii	XP_003680478.1	Fungi		LLQAYFLVADDMMDQSITRRGQP
Toxoplasma gondii	**ABG02859.1**	**Apicomplexa**		**LLQSCFLVMDDVMDHSLTRRGKQ**
Trametes versicolor FP-101664 SS1	EIW53149.1	Fungi		LLQAFFLVSDDMMDQSVTRRGQP
Tremella mesenterica DSM 1558	EIW67324.1	Fungi		LLQAYFLVADDMMDQSITRRGQP
Tribolium castaneum	NP_001164089.1	Metazoa		LLQGFFLVTDDIIDRSEMRRGMP
Trichoderma atroviride IMI 206040	EHK41700.1	Fungi		LLQAFFLVSDDIMDSSITRRGQP
Trichoderma reesei QM6a	EGR47729.1	Fungi		LLQAFFLVSDDIMDSSITRRGQP
Trichoderma virens Gv29-8	EHK26181.1	Fungi		LLQAFFLVSDDIMDSSITRRGQP
Trichomonas vaginalis G3	XP_001318110.1	Parabasalia		ILQASFLVADDLMDKSPLRRDKP
Trichophyton equinum CBS 127.97	EGE04630.1	Fungi		LLQAFFLVSDDIMDSSITRRGEP
Trichophyton rubrum CBS 118892	XP_003232123.1	Fungi		LLQAFFLVSDDIMDSSITRRGEP
Trichophyton tonsurans CBS 112818	EGD93061.1	Fungi	X	LLQAFFLVSDDIMGQLDHS---P
Trichophyton verrucosum HKI 0517	XP_003019208.1	Fungi		LLQAFFLVSDDIMDSSITRRGEP
Trichoplax adhaerens	XP_002116714.1	Metazoa		FLQAFFLVADDIMDDSVTRRGQP
Trichosporon asahii var. asahii CBS 8904	EKD02817.1	Fungi		--LGFFLVSDDLMDASITRRGQP
Trypanosoma brucei brucei strain 927/4 GUTat10.1	XP_845959.1	Kinetoplastida		FLQAHYLVEDDIMDNSVTRRGKP
Trypanosoma brucei gambiense DAL972	CBH12431.1	Kinetoplastida		FLQAHYLVEDDIMDNSVTRRGKP
Trypanosoma congolense IL3000	CCC91446.1	Kinetoplastida		FLQAHYLVEDDIMDKSLTRRGEP
Trypanosoma cruzi marinkellei	EKF39567.1	Kinetoplastida		FLQAHYLVEDDIMDSSVMRRGKP
Trypanosoma cruzi strain CL Brener	XP_813173.1	Kinetoplastida		FLQAHYLVEDDIMDGSVMRRGKP

Trypanosoma vivax Y486	CCC48988.1	Kinetoplastida		FLQAHYLVEDDIMDGSLTRRGMP
Tuber borchii	ABD66600.1	Fungi		LLQAMFLVADDIMDSSKTRRGSP
Tuber melanosporum Mel28	XP_002835078.1	Fungi		LLQGMFLVADDIMDSSKTRRGSP
Uncinocarpus reesii 1704	XP_002544446.1	Fungi		LLQAFFLVSDDMMDASITRRGEP
uncultured archaeon	CBH37355.1	Archaea?		LMQSYLLIHDDIMDEDELRRGKP
uncultured bacterium	EKD44047.1	Bacteria?		IFQTAILAHDDIIDKSPTRRGRP
uncultured bacterium	EKE18866.1	Bacteria?		FVHLFLLVHDDIIDRGDLRHGKE
uncultured bacterium	EKE19607.1	Bacteria?		LVHLFLLIHDDIIDRGDLRHGQL
Ustilago hordei	CCF53585.1	Fungi		LLQAYFLVADDMMDASVTRRGQP
Ustilago maydis 521	XP_757593.1	Fungi		LLQAYFLVADDMMDASVTRRGQP
Vanderwaltozyma polyspora DSM 70294	XP_001646858.1	Fungi		LLQAYFLVADDMMDKSITRRGQP
Verticillium albo-atrum VaMs.102	XP_003008565.1	Fungi		LLQAFFLVSDDIMDSSITRRGKP
Verticillium dahliae VdLs.17	EGY18196.1	Fungi		LLQAFFLVSDDIMDSSITRRGKP
Vitis vinifera	XP_002272641.1	Viridiplantae		WLQAYFLVLDDIMDNSHTRRGQP
Volvox carteri f. nagariensis	XP_002957674.1	Viridiplantae		WLQAFFLVADDIMDGSITRRGQP
Wallemia sebi CBS 633.66	EIM20287.1	Fungi		LLQAYFLVADDMMDGSITRRGQP
Wickerhamomyces ciferrii	CCH43512.1	Fungi		LLQAYFLVADDMMDKSITRRGQP
Withania somnifera	ADR10437.1	Viridiplantae	X	WLQAYFLVLDDIMDNSHTRPRSTN
Wolfiporia cocos	AFR13038.1	Fungi		FLQAFFLVSDDMMDQSVTRRGQP
x Citrofortunella microcarpa	AAK68152.1	Viridiplantae		WLQAYFLVLDDIMDGSHTRRGQP
Xenopus laevis	NP_001084626.1	Metazoa		LLQAFFLVADDIMDNSVTRRGQP
Xenopus laevis	NP_001090113.1	Metazoa		LLQAFFLVADDIMDNSVTRRGQP
Xenopus (Silurana) tropicalis	NP_001015867.1	Metazoa		LLQAFFLVADDIMDNSVTRRGQP
Yarrowia lipolytica	XP_503599.1	Fungi		LLQAFFLVSDDIMDESKTRRGQP
Zea mays	NP_001141329.1	Viridiplantae		WLQAYFLVLDDIMDNSQTRRGQP
Zea mays	**NP_001105039.1**	**Viridiplantae**		**WLQAFFLVLDDIMDDSHTRRGQP**
Zobellia galactanivorans	YP_004736392.1	Bacteria, Bacteroidetes		TFHNFSLVHDDIMDDAPLRRGKT
Zunongwangia profunda SM-A87	YP_003587050.1	Bacteria, Bacteroidetes		IFHNFSLVHDDIMDDAPLRRGKA
Zygosaccharomyces rouxii	XP_002498790.1	Fungi		LLQAYFLVADDMMDRSITRRGQP
Zymoseptoria tritici IPO323	XP_003850094.1	Fungi		LLQAFFLVSDDIMDSSKTRRGNP

* Sequences characterized as bifunctional FPP/GPPS are highlighted in gray and use bold font
* Excluded from CLD analysis (X in red) were the sequences that either did not present the canonical DDxxD FARM motif or had rare insertions (see main text)
