# Supplementary material for: Cloning and characterization of bifunctional enzyme farnesyl diphosphate/geranylgeranyl diphosphate synthase from Plasmodium falciparum
Source: Malar J. 2013 Jun 4;12:184. doi: 10.1186/1475-2875-12-184 (PMC3679732; doi:10.1186/1475-2875-12-184)
Supplement: Additional file 3 — Expression of the rPfFPPS. SDS-polyacrylamide gel 12% was stained with Coomassie Brilliant Blue. Lane 1, soluble fraction from extract of E. coli BL21(DE3) pLys RIL/rPfFFPS; Lane 2, rPfFPPS fused with GST; lane 3, GST. Molecular size standards are indicated on the left (kDa). [file 1475-2875-12-184-S3.pdf]

File 3. Expression of the rPfFPPS

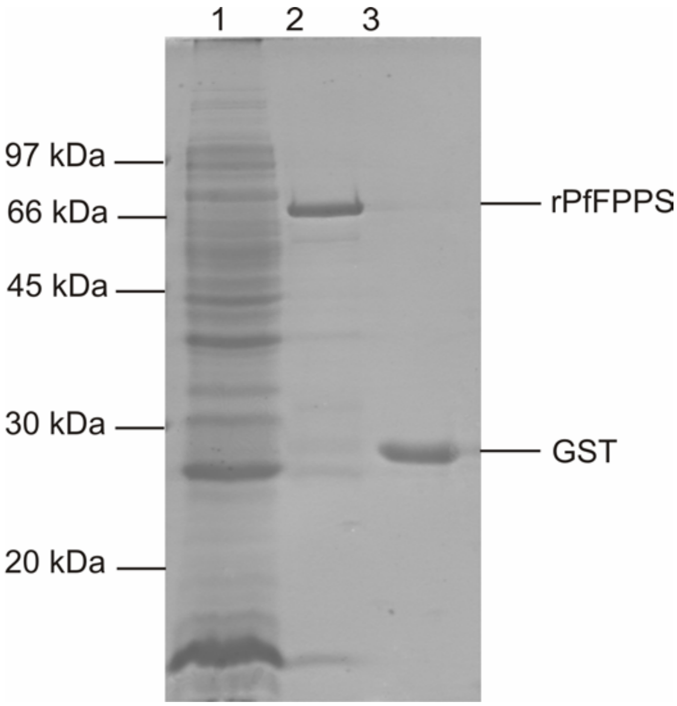

SDS-polyacrylamide gel 12% was stained with Coomassie Brilliant Blue. Lane 1, soluble fraction from extract of *E. coli* BL21(DE3) pLys RIL/rPfFFPS; Lane 2, rPfFPPS fused with GST; lane 3, GST. Molecular size standards are indicated on the left (kDa).
