# Supplementary material for: Cloning and characterization of bifunctional enzyme farnesyl diphosphate/geranylgeranyl diphosphate synthase from Plasmodium falciparum
Source: Malar J. 2013 Jun 4;12:184. doi: 10.1186/1475-2875-12-184 (PMC3679732; doi:10.1186/1475-2875-12-184)
Supplement: Additional file 4 — MM plots of the steady-state initial velocity experiments for rPfFPPS/GGPPS. R2 values for each plot are: A) 0.99; B) 0.99; C) 0.98; D) 0.99; E) 0.97; F) 0.99. Experiments and data analysis were conducted as detailed under Methods, rPfFPPS kinetic assays section. Concentration ranges of each varied substrate are depicted on Table 1. Data were fitted to Equation (1). [file 1475-2875-12-184-S4.pdf]

File 4. MM plots of the steady-state initial velocity experiments for rPfFPPS/GGPPS

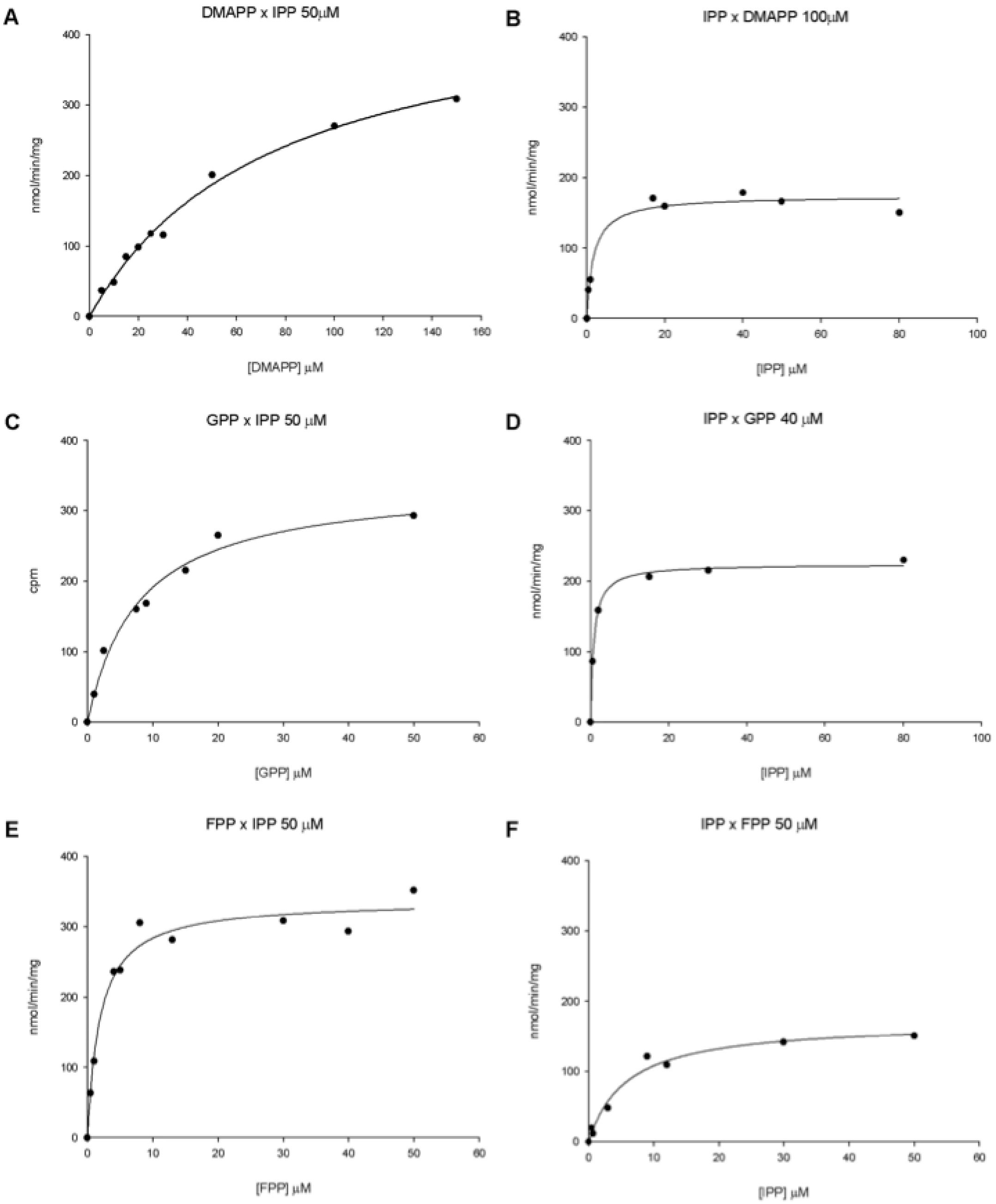

R^2 values for each plot are: **A**) 0.99; **B**) 0.99; **C**) 0.98; **D**) 0.99; **E**) 0.97; **F**) 0.99. Experiments and data analysis were conducted as detailed under **Methods**, *rPfFPPS kinetic assays* section. Concentration ranges of each varied substrate are depicted on Table 1. Data were fitted to **Equation (1)**.
