# Supplementary material for: Cloning and characterization of bifunctional enzyme farnesyl diphosphate/geranylgeranyl diphosphate synthase from Plasmodium falciparum
Source: Malar J. 2013 Jun 4;12:184. doi: 10.1186/1475-2875-12-184 (PMC3679732; doi:10.1186/1475-2875-12-184)
Supplement: Additional file 5 — Inhibition of rPfFPPS/GGPPS activity by risedronate. A) Substrate pair FPP/IPP (R2 = 0.98); B) Substrate pair GPP/IPP (R2 = 0.99). rPfFPPS is expressed as its fractional activity; and risedronate concentrations were plotted on log scale. Data were fitted to Equation (2). [file 1475-2875-12-184-S5.pdf]

**File 5.** Inhibition of rPfFPPS/GGPPS activity by risedronate

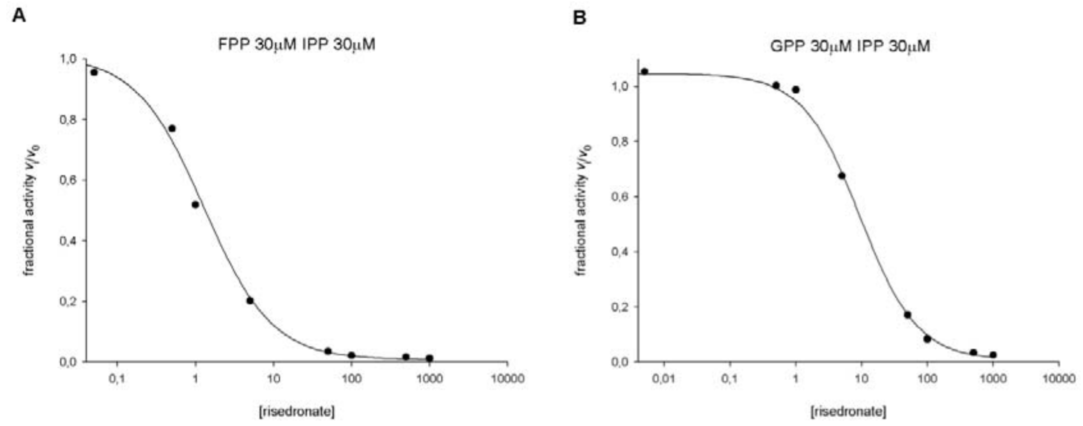


**A)** Substrate pair FPP/IPP ($R^2$ = 0.98); **B)** Substrate pair GPP/IPP ($R^2$ = 0.99). rPfFPPS is expressed as its fractional activity; and risedronate concentrations were plotted on log scale. Data were fitted to **Equation (2)**.
