# Supplementary material for: Cloning and characterization of bifunctional enzyme farnesyl diphosphate/geranylgeranyl diphosphate synthase from Plasmodium falciparum
Source: Malar J. 2013 Jun 4;12:184. doi: 10.1186/1475-2875-12-184 (PMC3679732; doi:10.1186/1475-2875-12-184)
Supplement: Additional file 6 — Sequence logo analysis of the chain-length determination region. All sequences containing the canonical DDxxD FARM motif were submitted to sequence logo analysis, as described in the Methods section. Total height of each position reflects overall sequence conservation at that column; height of each residue in a column reflects its proportion in relation to other possible residues for that column. Colors are for clarity, with aspartate in red, aromatic amino acids in blue, serine and cysteine in cyan, and all other amino acids in black. [file 1475-2875-12-184-S6.pdf]

File 6. Sequence logo analysis of the chain-length determination region

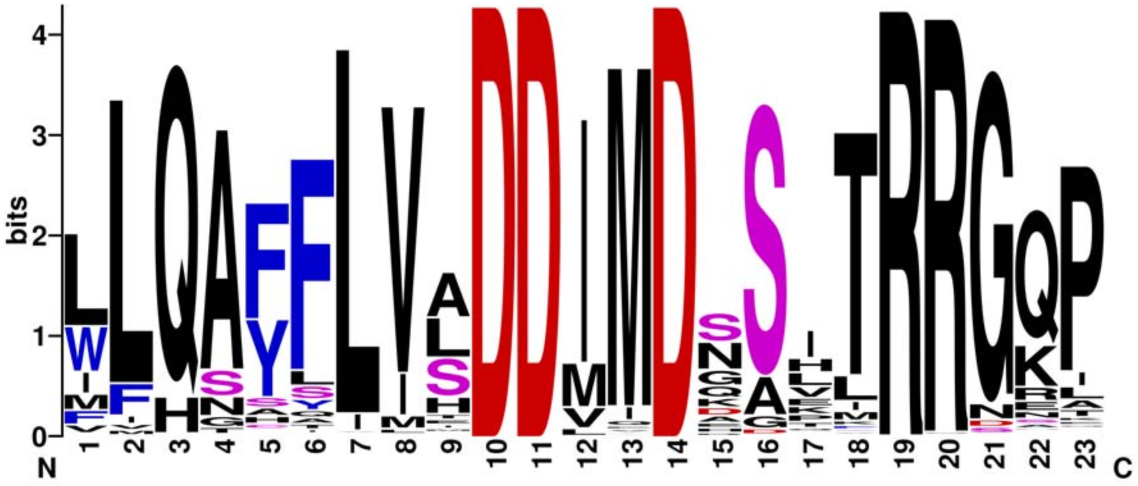

All sequences containing the canonical DDxxD FARM motif were submitted to sequence logo analysis, as described in the Methods section. Total height of each position reflects overall sequence conservation at that column; height of each residue in a column reflects its proportion in relation to other possible residues for that column. Colors are for clarity, with aspartate in red, aromatic amino acids in blue, serine and cysteine in cyan, and all other amino acids in black.
