# Supplementary material for: Cloning and characterization of bifunctional enzyme farnesyl diphosphate/geranylgeranyl diphosphate synthase from Plasmodium falciparum
Source: Malar J. 2013 Jun 4;12:184. doi: 10.1186/1475-2875-12-184 (PMC3679732; doi:10.1186/1475-2875-12-184)

File 7. Proposed kinetic mechanism for rPfFPPS. GGPP synthesis is proposed to follow a bi-bi ordered mechanism in an intricate system of parallel and consecutive reactions

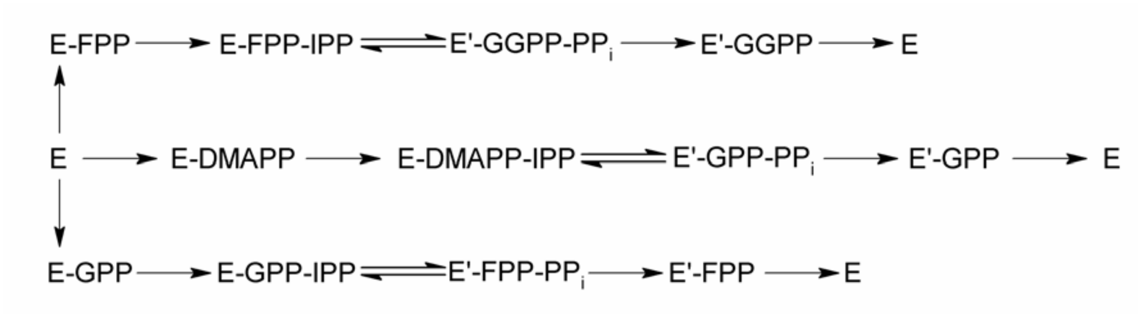

Supplement: Additional file 7 — Proposed kinetic mechanism for rPfFPPS. GGPP synthesis is proposed to follow a bi-bi ordered mechanism in an intricate system of parallel and consecutive reactions. [file 1475-2875-12-184-S7.pdf]
